# Prediction of Vancomycin Area Under the Curve With Trough Concentrations Only: Performance Evaluation of Pediatric Population Pharmacokinetic Models

**DOI:** 10.1093/infdis/jiaf059

**Published:** 2025-02-04

**Authors:** Stef Schouwenburg, Tim Preijers, Robert B Flint, Enno D Wildschut, Birgit C P Koch, Brenda C M de Winter, Alan Abdulla

**Affiliations:** Department of Hospital Pharmacy, Erasmus University Medical Centre, Rotterdam, the Netherlands; Rotterdam Clinical Pharmacometrics Group, Erasmus University Medical Centre, Rotterdam, the Netherlands; Department of Hospital Pharmacy, Erasmus University Medical Centre, Rotterdam, the Netherlands; Rotterdam Clinical Pharmacometrics Group, Erasmus University Medical Centre, Rotterdam, the Netherlands; Department of Hospital Pharmacy, Erasmus University Medical Centre, Rotterdam, the Netherlands; Rotterdam Clinical Pharmacometrics Group, Erasmus University Medical Centre, Rotterdam, the Netherlands; Department of Neonatal and Pediatric Intensive Care, Division of Neonatology, Erasmus University Medical Centre-Sophia Children’s Hospital, Rotterdam, the Netherlands; Department of Neonatal and Pediatric Intensive Care, Division of Pediatric Intensive Care, Erasmus University Medical Centre–Sophia Children's Hospital, Rotterdam, the Netherlands; Department of Hospital Pharmacy, Erasmus University Medical Centre, Rotterdam, the Netherlands; Rotterdam Clinical Pharmacometrics Group, Erasmus University Medical Centre, Rotterdam, the Netherlands; Center for Antimicrobial Treatment Optimization Rotterdam (CATOR), Rotterdam, the Netherlands; Department of Hospital Pharmacy, Erasmus University Medical Centre, Rotterdam, the Netherlands; Rotterdam Clinical Pharmacometrics Group, Erasmus University Medical Centre, Rotterdam, the Netherlands; Center for Antimicrobial Treatment Optimization Rotterdam (CATOR), Rotterdam, the Netherlands; Department of Hospital Pharmacy, Erasmus University Medical Centre, Rotterdam, the Netherlands; Rotterdam Clinical Pharmacometrics Group, Erasmus University Medical Centre, Rotterdam, the Netherlands

**Keywords:** bayesian estimation, model-informed precision dosing, pharmacokinetics, therapeutic drug monitoring, vancomycin

## Abstract

**Background:**

In pediatric patients, vancomycin plays a pivotal role in combating infections, necessitating precise therapeutic drug monitoring to ensure efficacy and safety. The adoption of model-informed precision dosing (MIPD) has demonstrated potential in optimizing dosing strategies based on the 24-hour area under the concentration-time curve (AUC_24h_). However, the predictive performance of population pharmacokinetic models by using only trough concentrations to estimate AUC_24h_ has not been evaluated.

**Methods:**

The predictive performance of 23 vancomycin population pharmacokinetic models was retrospectively evaluated in 2 cohorts: cohort A, 21 patients with postnatal age <50 days; cohort B, 124 patients with postnatal age ≥50 days. Multiple scenarios were investigated by using peak and trough concentration, peak concentration solely, trough concentration solely, or covariate information (a priori). The median AUC_24h_ per patient across all models was used as the true AUC_24h_.

**Results:**

For both cohorts, the relative root mean square error (rRMSE) for the AUC_24h_ precision based on trough concentrations was similar to the rRMSE based on a peak and trough sample. For cohort A, the models by Chen, Colin, and Mehrotra showed the best trough-based performance with the lowest relative bias (range, –3.3% to –2.6%) and rRMSE (range, 6.8%-7.3%). For cohort B, the models from Alsultan and Lv illustrated the lowest relative bias (range, 1.75% to –5.4%) and rRMSE (range, 16.6%-15.1%).

**Conclusions:**

This study illustrates that trough concentration–based AUC_24h_ estimation is a feasible approach in vancomycin MIPD. These findings endorse the selected models for advanced MIPD vancomycin therapy in pediatrics, although further investigation into clinical outcomes is recommended.

## Teaser

Trough-based 24-hour area under the concentration-time curve estimation is feasible for pediatric vancomycin therapeutic drug monitoring by using model-informed precision dosing. Evaluation of 23 models revealed that the Chen, Colin, and Mehrotra models for postnatal age <50 days and the Alsultan and Lv models for postnatal age ≥50 days showed the best performance in vancomycin trough-based model-informed precision dosing.

Vancomycin is a glycopeptide antibiotic that has widespread use in the pediatric population, especially in patients who are critically ill. Over the past decade, multiresistant bacterial cultures, particularly methicillin-resistant *Staphylococcus aureus* (MRSA), have become endemic in pediatric intensive care units, leading to increased transmission and invasive hospital-acquired infections [1, 2]. Vancomycin is widely used for the prevention and treatment of MRSA infections in pediatric patients [3].

To ensure the efficacy and safety of vancomycin, therapeutic drug monitoring (TDM) is essential [4]. As illness-related pathophysiologic alterations cause changes in individual pharmacokinetic (PK) parameters, TDM is particularly challenging in neonatal and pediatric patients. As a consequence, inadequate vancomycin treatment may result in drug toxicity, often nephrotoxicity, or subtherapeutic exposure [5, 6]. The vancomycin PK/pharmacodynamic index for optimal exposure is the 24-hour area under the concentration-time curve (AUC_24h_) over the minimum inhibitory concentration (MIC) of the targeted pathogen (AUC_24h_/MIC_1 mg/L_). Until the 2020 consensus guideline, TDM based on vancomycin trough levels (10–15 mg/L) had been applied in some countries as the surrogate target accommodating adequate AUC_24h_ exposure [4, 6]. To improve antibiotic exposure and ultimately patient outcomes, the updated consensus review recommends calculating the AUC_24h_ and targeting it to 400 to 600 mg·h/L instead of dosing by trough levels.

Ideally, the vancomycin AUC_24h_ target of 400 to 600 mg·h/L should be obtained by maximum a posteriori bayesian estimation with software such as MwPharm++ or InsightRx Nova. Recent advancements in bayesian estimation, enabling model-informed precision dosing (MIPD) of vancomycin in adult patients, have led to more widespread application of the AUC_24h_ as an end point for vancomycin dosing in adults [4]. Models for different patient populations are available within MIPD software tools, containing various patient characteristics that need to be taken into account to select an appropriate population PK (popPK) model. External evaluation of the predictive performance from existing popPK models for vancomycin has shown that it is possible to accurately estimate the individual PK parameters for vancomycin in adults who are critically ill with measured vancomycin concentrations for TDM [7].

Improvements to vancomycin exposure may be achieved through MIPD based on patient characteristics and measured blood samples. The sampling strategy for traditional vancomycin dose adaptations relies on the interpretation of trough concentrations. However, for accurate estimation of the AUC_24h_, it is advised to obtain at least 1 postdistributional peak and 1 trough vancomycin concentration at the end of a dosing interval. The ability to accurately determine a trough-based AUC_24h_ without a peak concentration offers advantages such as improved clinical feasibility and reduced patient burden. In this study, we aim to retrospectively evaluate the accuracy and precision of trough-based AUC_24h_ estimation using existing popPK models for vancomycin and TDM blood samples for pediatric patients.

## METHODS

### Study Approval

The Medical Ethics Committee of the Erasmus University Medical Centre granted the study a waiver for informed consent (MEC-2023-0054) because the retrospective analysis of anonymized patient data was assessed to involve minimal risks to the identified participants.

### Patient Data Collection

This retrospective PK study collected patient demographic and clinical data from patients admitted to the Erasmus University Medical Centre–Sophia Children’s Hospital between January 2017 and July 2023. Patients with a predose peak and trough sample available within the same day were identified by the hospital's drug concentration reporting software LabTrain (version 3.48.1.6; Bodegro). After patient selection, data were collected per the electronic patient records. Patients were eligible for inclusion if they were <18 years old on the day of sampling, had a gestational age >25 weeks, and received intermittent intravenous vancomycin therapy. Patients were excluded when receiving extracorporeal membrane oxygenation, continuous venovenous hemofiltration, or vancomycin by continuous infusion. The data described vancomycin dose administration times, dosage amounts, laboratory results (measured serum concentrations of creatinine), collection times and measured concentrations of vancomycin in plasma, age, body weight (at the time of vancomycin measurement), height, and sex. Patient data that appeared to be mistakenly entered were removed from analysis (eg, incorrect collection times or medication administration times or quantities). The final data set was divided into 2 cohorts. Cohort A consisted of patients with a postnatal age <50 days, including premature patients. Cohort B included patients with a postnatal age ≥50 days. This arbitrary age threshold was chosen since most population models for the youngest pediatric patients include those up to 2 months of age.

### PopPK Model Selection

PopPK models were selected by using the systematic review by Chung et al [8]. Furthermore, a literature search in PubMed was conducted to identify popPK models describing vancomycin concentrations in pediatric patients that were published after the review from Chung et al. Models were excluded if based on <25 patients and requiring covariates that were unavailable in our data set. Corresponding authors were contacted to provide their model code, or models were extracted from their corresponding publications. The selected models were encoded in NONMEM version 7.4 (Icon Development Solutions).

### Model Evaluations

Patient data were extrapolated to simulate steady state pharmacokinetics. Four scenarios were evaluated for each patient: a peak and trough sample, a peak sample separately, a trough sample separately, or provision of covariate information solely (a priori dosing). For each patient, the median AUC_24h_ value, estimated from all selected popPK models by a peak and trough concentration, was used to represent the true AUC_24h_.

### Performance Metrics

The model fit was evaluated by visual predictive checks and goodness-of-fit plots (GOFs; observed vs predicted vancomycin concentration) performed with Perl-speaks-NONMEM version 4.2.0, Pirana software version 3.0.0 (Certara), and R version 4.1.2 (nonmem2R package; R Foundation for Statistical Computing). Predictive performance was assessed by comparing the predicted (a priori dosing) and estimated (trough and/or peak sample) vancomycin AUC_24h_. Performance was assessed by a graphical and descriptive approach. The relative bias (rBias) and relative root mean square error (rRMSE) were used to determine the accuracy and precision of the estimated parameters, respectively. The performance of the models was considered clinically acceptable when the rBias was between −15% and 15% [9, 10]. Additionally, the precision metric (rRMSE) should be as small as possible, preferably <20%. The rRMSE and rBias were calculated by the following equations:


$$rBias=\frac{1}{n}\;\times \overset{i}{\underset{1}{\sum }}\;\left(\frac{{\left(predicted_{i}-true_{i}\right)}^{2}}{true_{i}^{2}}\right)\;\times 100$$

$$rRMSE=\;\sqrt{\frac{1}{n}\times \overset{i}{\underset{1}{\sum }}\;\left(\frac{{\left(predicted_{i}-true_{i}\right)}^{2}}{true_{i}^{2}}\right)}\;\times 100.$$

## RESULTS

### Demographics

Cohort A consisted of 21 patients with a median age of 17 days (range, 3–40) and gestational age of 37.1 weeks (range, 25.4–41.0). Cohort B consisted of 124 patients with a median age of 1.8 years (range, 0.1–17.2). The median of all vancomycin concentrations was 17.1 mg/L (range, 5.3–56.2) for cohort A and 17.4 mg/L (range, 0.8–76.7) for cohort B. See Table 1 for patient demographics and Table 2 for sampling and dosage information.

**Table 1.** Baseline Characteristics

	Median (Range) or %	
	Cohort A <50 d (n = 21)	Cohort B ≥50 d (n = 124)
Female, %	57.1	63.8
Weight, kg	2.5 (0.7–4.2)	12.2 (2.3–74.6)
Age, y	…	1.8 (0.1–17.2)
Gestational age, wk	37.1 (25.4–41.0)	…
Postnatal age,^a^ d	17 (3–40)	671 (50–6273)
Postmenstrual age, wk	39.7 (27.9–44.9)	…
Dose, mg	35 (5.5–80)	175 (20–1200)
Dose, mg/kg	12.0 (5.8–26.9)	14.9 (5.8–33.5)
Serum creatinine, µmol/L	29 (15–81)	19 (6–103)
Creatinine clearance, mL/min/1.73 m^2^	…	148.9 (36–438)
Premature,^b^ %	47.6	…

^a^Postnatal age during therapy. Creatinine clearance was calculated per the Boer et al method for patients with a postnatal age <365 days and per the Schwartz bedside equation for patients with a postnatal age >365 days [11, 12].

^b^Premature: gestational age <37 weeks.

**Table 2.** Sampling and Dosing Characteristics

	Median (Range; IQR)	
	Postnatal Age <50 d (n = 21)	Postnatal Age ≥50 d (n = 124)
No. of plasma concentrations		
Trough	21	124
Peak	21	124
Plasma concentration, mg/L		
Trough	13.3 (5.3–48.5)	8.9 (0.8–30.5)
Peak	27 (14.3–56.2)	30.8 (13.2–76.7)
Time after dose samples, h		
Trough	6.22 (4.34–26.77)	6.27 (4.32–41.53)
Peak	1.41 (0.12–5.91)	2.04 (0.16–21.15)
No. of dosages before first sample measurement	11 (1–29; 3–16)	12 (1–184; 8–18)

### PopPK Models

The model exclusion process is described in the supplementary material. Subsequently, 13 popPK models for cohort A [13–25] and 12 popPK models for cohort B [26–37] were selected for evaluation (Supplementary Tables 1–4). All popPK models for cohort A were structurally identical (1-compartmental), while 2 of the popPK models for cohort B were 2-compartmental. The majority of cohort A popPK models comprised postmenstrual age (12/13), body weight (12/13), and/or serum creatinine (9/13) as covariate relationships associated with vancomycin clearance (9/13). All models for cohort B contained body weight as a covariate relationship with vancomycin clearance. Serum creatinine (3/12) and creatinine clearance (3/12) were included as covariates on renal function.

### Model Evaluation

The visual predictive checks (Supplementary Figures 1 and 2) suggested a varying predictive performance among the popPK models when the peak and trough concentrations were used. Adequate model fit was supported by the GOFs (Supplementary Figures 3–6). The GOFs illustrate adequate prediction of trough concentrations among most models. The models illustrate difficulty predicting peak concentrations, demonstrating increased residual error in the GOFs among higher concentrations. The median true AUC_24h_ was 535 (IQR, 463–669; range, 339–1183) for cohort A and 566 (IQR, 489.25–680.75; range, 320–2132) for cohort B. Overall, the trough and peak sample scenario performed best when compared with the true AUC_24h_ among all models.

**Figure 1.**
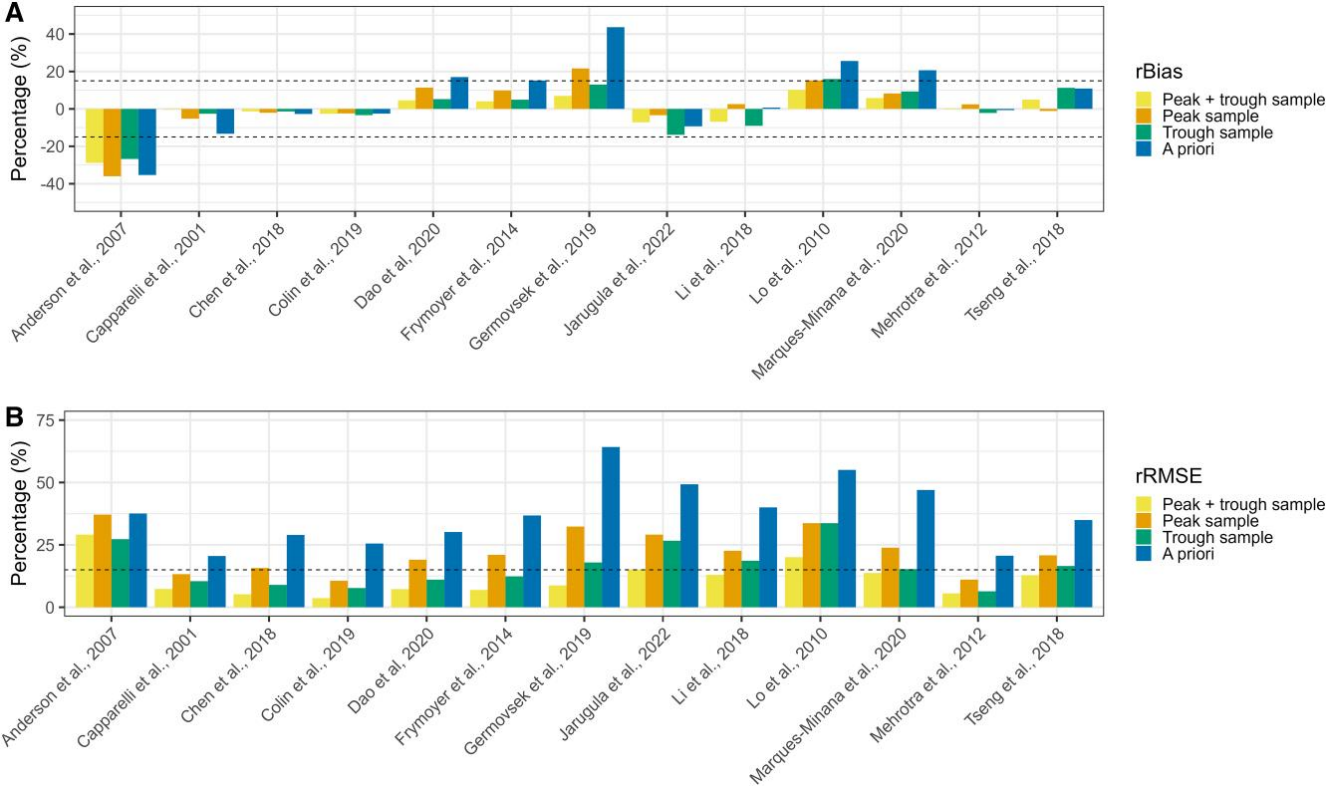
The predicted AUC_24h_ vs the median true AUC_24h_ for all models for each patient: *A*, relative bias (rBias); *B*, relative root mean squared error (rRMSE). The median true AUC_24h_ is based on the peak and trough sample for cohort A (postnatal age <50 days). The 4 scenarios are as follows: a peak and trough sample, a peak sample, a trough sample, and provision of covariate information solely (a priori). Dashes lines: *A*, +15% and –15% threshold for rBias; *B*, +20% threshold for rRMSE. AUC_24h_, 24-hour area under the concentration-time curve.

In cohort A, a range from –35.9% to 43.7% for rBias and from 3.6% to 64.2% for rRMSE across all models was obtained (Figure 1). The rBias and rRMSE of the a priori scenario were higher than all other scenarios. When the true AUC_24h_ was compared with an AUC_24h_ based on trough sampling, the models of Chen et al, Colin et al, and Mehrotra et al [22, 23, 25] seemed to perform best. For these models, rBias and rRMSE ranged between –3.3% and –1.4% and between 6.4% and 9.0%, respectively (Supplementary Table 5).

**Figure 2.**
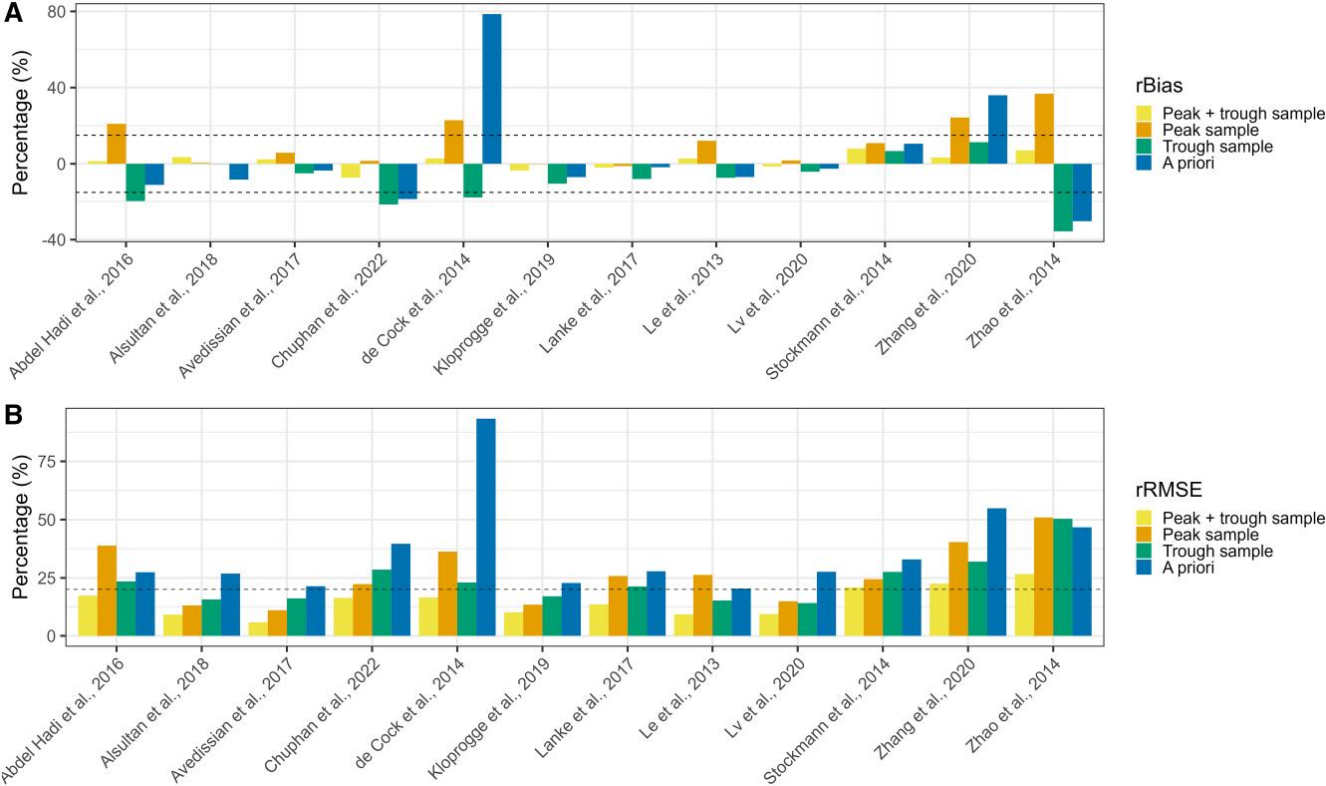
The predicted AUC_24h_ vs the median true AUC_24h_ for all models for each patient: *A*, relative bias (rBias); *B*, relative root mean squared error (rRMSE). The median true AUC_24h_ is based on the peak and trough sample for cohort B (postnatal age ≥50 days). The 4 scenarios are as follows: a peak and trough sample, a peak sample, a trough sample, and provision of covariate information solely (a priori). Dashes lines: *A*, +15% and –15% threshold for rBias; *B*, +20% threshold for rRMSE. AUC_24h_, 24-hour area under the concentration-time curve.

The rBias and rRMSE of the evaluated popPK models for cohort B ranged from –35.6% to 78.6% and from 5.8% to 93.3%, respectively (Figure 2). As illustrated for cohort A, the rBias and rRMSE of the a priori scenario were higher than all other scenarios. Overall, a trough sample yielded a lower rBias and rRMSE in comparison with a peak sample (median rBias, –7.4% vs 8.3%; median rRMSE, 21.3% vs 24.4%). The best-performing popPK models were those of Alsultan et al and Lv et al, with an rBias and rRMSE ranging from –0.1% to –4.2% and from 15.6% to 14.1% [32, 36]. All numerical values for the rBias (with 95% CI) and RMSE are shown in Supplementary Tables 5 and 6. The numerical differences from the true AUC_24h_ in rBias and rRMSE are visualized in Supplementary Figures 7 and 8.

**Figure 3.**
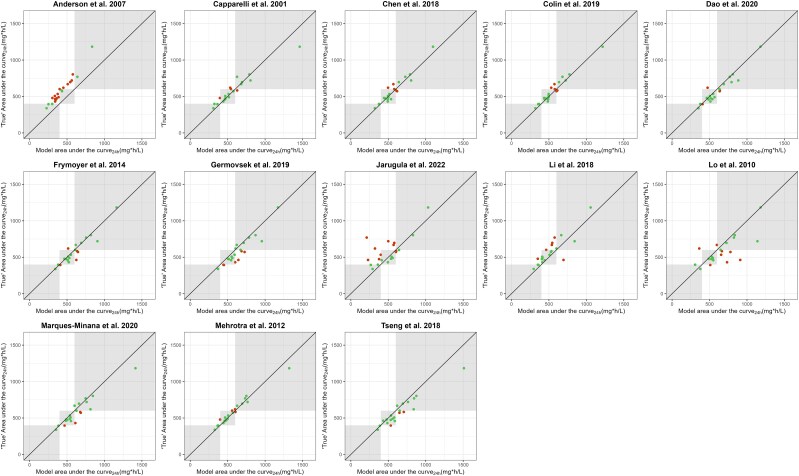
Model vancomycin AUC_24h_ based on trough concentration vs true AUC_24h_ for cohort A (postnatal age <50 days). Plots present the 13 evaluated population pharmacokinetic models. Green dots in the gray shaded area illustrate individual AUC_24h_ values that show alignment in dosage advice based on the model AUC_24h_ and true AUC_24h_. Red dots illustrate patients requiring a dosage change based on the model AUC_24h_ or true AUC_24h_. For example, a green dot in the left-most gray window indicates a dosage increase by the model AUC_24h_ and the true AUC_24h_ (<400 mg·h/L). AUC_24h_, 24-hour area under the concentration-time curve.

**Figure 4.**
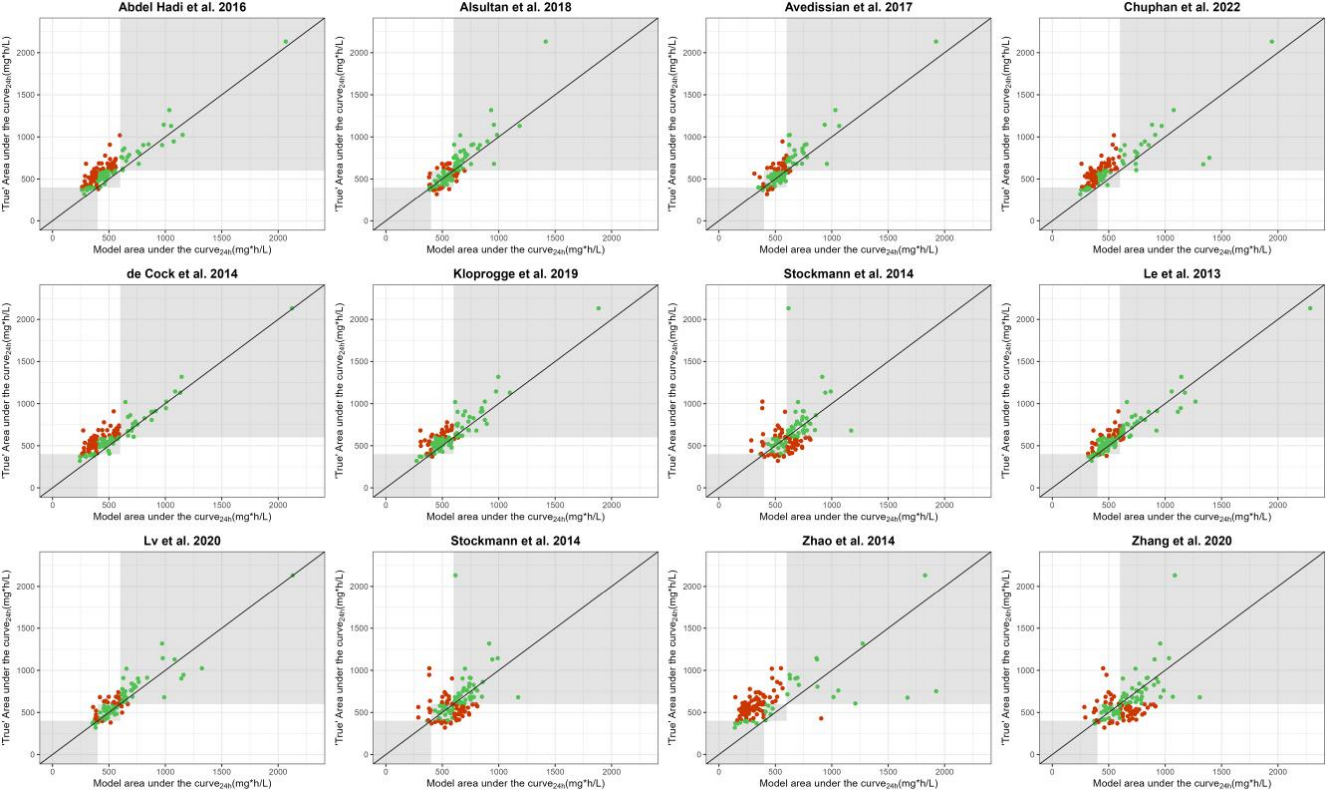
Model vancomycin AUC_24h_ based on trough concentration vs true AUC_24h_ for cohort B (postnatal age ≥50 days). Plots present the 13 evaluated population pharmacokinetic models. Green dots in the gray shaded area illustrate individual AUC_24h_ values that show alignment in dosage advice based on the model AUC_24h_ and true AUC_24h_. Red dots illustrate patients requiring a dosage change based on the model AUC_24h_ or true AUC_24h_. For example, a green dot in the left-most gray window indicates a dosage increase by the model AUC_24h_ and true AUC_24h_ (<400 mg·h/L). AUC_24h_, 24-hour area under the concentration-time curve.

In Figures 3 and 4, the model-predicted vancomycin AUC_24h_ based on a trough concentration is plotted vs the true AUC_24h_ to illustrate alignment in dosage advice (compliance of AUC_24h_ within 400- to 600-mg·h/L window). The models of Chen et al, Mehrotra et al, and Colin et al [22, 23, 25] demonstrated the highest percentage of patients not requiring dosage adjustment (76.2%, 76.2%, and 81.0%, respectively). In cohort B, performance for the models of Alsultan et al and Lv et al was good, as illustrated by the highest percentages not requiring a dosage adjustment and by the good compliance between the trough-estimated AUC_24h_ and true AUC_24h_ (73.8% and 77.0%). The percentages requiring dosage adjustment are shown in Supplementary Tables 7 and 8.

### Evaluation of Predicted Area Under the Curve

For each model, the AUC_24h_ was predicted a priori with covariates solely and estimated by available vancomycin concentrations per bayesian analysis. Supplementary Figures 9 and 10 illustrate the AUC_24h_ across all scenarios (peak and/or trough, a priori). The estimated AUC_24h_ based on trough samples ranged from 360.0 to 817.8 mg·h/L in cohort B (10th–90th percentile of the median estimated AUC_24h_ ). The model from Chen et al [22], which illustrated relatively low bias and precision, demonstrated similar ranges for the trough and median true AUC_24h_ (377–790 vs 397–770 mg·h/L, 10th–90th percentile).

## DISCUSSION

This study provides an overview of the available pediatric vancomycin popPK models and their ability to accurately predict the vancomycin AUC_24h_ based on patient covariate information and measured vancomycin concentrations (peak and/or trough samples). As rBias illustrates a model’s trend to over- or underestimation, predictive performance varies more widely across all models. The rRMSE for the popPK models illustrates a comparable profile due to its demonstration of magnitude of errors. A peak and trough concentration was most valuable, as for almost all models, accuracy and precision improved when compared with the single trough or peak concentration or a priori prediction. In some models, a single trough concentration performed almost equal to the 2-sample scenario (peak/trough). For cohort A, a more distinct preference toward the single trough concentration scenario can be argued since it performed visibly better at estimating the true AUC_24_. In cohort B, this effect is less clearly visible. However, the single trough scenarios performed similar to the single peak scenario. This result might be explained by the decrease in interindividual variability in the pediatric population once the neonatal stage is over. During the first year of life, renal function matures to 90% of the adult value, while the first weeks of life are subject to significant changes in distribution volume [38, 39]. This study, powered by real-world data, illustrates that a trough-based AUC_24h_ estimation does not underperform in the pediatric population as compared with the true AUC_24h_ estimation. Our findings indicate that appropriate model selection is crucial when relying on a trough concentration solely and could introduce imprecision since addition of a peak sample may improve rBias and rRMSE. Additionally, the results illustrate wide variability in model performance with the current data, highlighting the importance of external validation of published popPK models prior to implementation for MIPD. It is illusory that a single popPK model can satisfy adequate model performance across the entire age range, as heterogeneity is specific to the pediatric population (eg, critical illness, development). In populations for which blood draws are challenging (eg, neonates), a trough concentration is sufficient to estimate the AUC_24h_. Yet, in special patient populations requiring high care, a peak and trough concentration may be appropriate.

The switch from trough-only to AUC-guided monitoring was mainly adopted to maintain efficacy against MRSA infections while lowering the risk of vancomycin-associated acute kidney injury [3]. It is believed that using a bayesian approach to estimate the AUC_24h_ is ideally performed with 2 steady state vancomycin concentrations: 1 peak concentration (1 hour after infusion) and 1 trough concentration at the end of the dosing interval. Ideally, only a trough sample would be required, especially since performing vancomycin TDM is challenging in pediatric patients who are critically ill, due to blood volume restrictions. Discordance between the AUC_24h_ and trough may result in decreased therapy efficacy due to a supra- or subtherapeutic AUC_24h_ despite trough levels within the therapeutic window. Kishk et al demonstrated that a trough-based approach to achieve an AUC_24h_ >400 mg·h/L was variable when based on the applied calculation method [40]. However, multiple studies describe adequate correlation between vancomycin trough concentrations and AUC_24h_ in pediatric patients without using a bayesian approach [41]. Notably, it was demonstrated that lower trough concentrations (>10 mg/L) achieved the target AUC_24h_ >400 mg·h/L [41, 42]. In contrast to these studies, Brandon et al suggest poor correlation between trough concentrations and AUC_24h_, indicating that a trough concentration may not serve as a surrogate marker [43]. In comparison, our study suggests that applying a bayesian approach for trough-based AUC_24h_ estimation via MIPD holds promise for improving vancomycin exposure. Applying trough-based AUC_24h_ estimation barely lowers precision (rRMSE) when compared with the peak and trough predictive performance.

The examined popPK models varied in covariate relationships, model structure, and estimates of population parameters. This variability could stem from the heterogeneity within the populations that were used to develop these models. The best-performing models of cohort A included weight, postmenstrual age, and serum creatinine as covariates on vancomycin clearance. In cohort B, Alsultan et al and Lv et al included only weight on vancomycin clearance as a covariate [32, 36]. These results indicate that body weight is an important covariate to explain the maturation of clearance and distribution volume. Hughes et al found the popPK models of Dao et al and Jacqz-Aigrain et al to be the most informative, resulting in predicted concentrations within 2.5 mg/L of the measured concentrations, confirming their suitability for MIPD [15, 44]. The same research group illustrated the benefit of a continuous learning framework, improving upon the appliance of MIPD standard of care in terms of predictive performance and AUC_24h_ estimation [45]. It is important for clinicians to exercise caution when applying popPK models created for specific patient groups to the broader hospital patient population, as the applicability of these models may not extend equally across different groups. An example of this may be the model by Anderson et al [13] in an extreme study population (preterm neonates) that underpredicts the PK profile of the neonates presented in this study (median rBias for all scenarios, –32.3%). Additionally, models based on smaller cohorts may not be able to capture a complete description of the popPK of vancomycin. The Colin et al model was developed by combining 4 data sets (561 patients) but was not externally validated [23]. The combination of multiple data sets might promote clinical use in a widespread population, extensively capturing population estimates, but will not translate to models that are transferable to new patient populations.

This research is subject to several limitations. Prospective dense sampling was not an option in the current cohorts, resulting in the prediction of the true AUC_24h_ based on the available peak and trough sample. It is undebatable that models demonstrating worse performance biased the true AUC_24h_, but this effect was minimalized by using the median across all models. Another way that this could have been prevented is by using the median weighted AUC_24h_ according to model fit. Additionally, the analysis was performed with a limited sample size; therefore, the results should be interpreted with caution but may serve as a proof of principle. Yet, error of retrospective time registration during peak concentrations may have a considerable influence on clearance. The inverse proportionality of the AUC_24h_ with clearance provides that a 5-minute uncertainty in time registration may cause bias and imprecision [46]. Additionally, obtaining a peak concentration measurement is not standard clinical practice in our institution, thus introducing bias into the cohorts, since the patients may be prone to lower vancomycin concentrations overall. Our cohorts comprised data from a single center, resulting in less heterogeneity. However, patients from all wards of Sophia Children's Hospital were included. There was also variability in the number of dosages before first sample measurement (median [range]: younger cohort, 11 [1–29]; older cohort, 12 [1–184]). To counter this, we extrapolated patient characteristics to simulate steady state pharmacokinetics. Since MIPD applies only popPK models for a specific population, popPK model extrapolation was, as much as possible, partially prevented by creating 2 cohorts. We acknowledge that further distinction could have been made within cohorts A and B, such as prematurity, as it is a large population group with variable PK parameters.

In conclusion, several models were evaluated on performance and seemed appropriate for vancomycin MIPD in hospitalized neonates and children. The popPK models with the best predictive performance based on a single trough concentration per patient (lowest rBias or rRMSE) included weight, postmenstrual age, and serum creatinine as covariates for cohort A (<50 days). For cohort B (≥50 days), weight was included as a covariate. Estimation of the AUC_24h_ with only a priori covariate information is useful for initial dosage advice prior to measuring blood concentrations. However, more adequate AUC_24h_ values were predicted with at least 1 vancomycin concentration as compared with a priori dosing. In all models, a peak and trough sample outperformed a trough sample in predicting the AUC_24h_. Yet, the precision (rRMSE) remained adequate. This study illustrates that trough-based AUC_24h_ estimation does not underperform in the pediatric population as compared with the true AUC_24h_ estimation. Trough-based AUC_24h_ estimation improves clinical feasibility of vancomycin TDM and decreases patient burden in children. The most accurate popPK models may serve as the cornerstone for MIPD in pediatric vancomycin therapy and strengthen their prioritization over alternative models. Yet, further clinical outcome studies are warranted.

## Supplementary Material

jiaf059_Supplementary_Data

## Supplementary Data

Supplementary materials are available at *The Journal of Infectious Diseases* online (http://jid.oxfordjournals.org/). Supplementary materials consist of data provided by the author that are published to benefit the reader. The posted materials are not copyedited. The contents of all supplementary data are the sole responsibility of the authors. Questions or messages regarding errors should be addressed to the author.

## Notes

***Author contributions.*** S. S., T. P., E. D. W., R. B. F., B. C. P. K., B. C. M. d. W., and A. A. made substantial contributions to conception and design of the PK trial, interpretation of data, and funding acquisition. S. S. and T. P. were responsible for data analysis. S. S. and T. P. directly accessed and verified the underlying data presented here. S. S. drafted the manuscript. S. S., T. P., E. D. W., R. B. F., B. C. P. K., B. C. M. d. W., and A. A. provided a critical revision for important intellectual content. All authors approved the final version to be published and agreed to be accountable for all aspects of the work. S. S. and A. A. had full access to all the data in the study and take responsibility for data integrity and accuracy of the data analysis.

***Disclaimer.*** The funders had no role in the collection, analysis, and interpretation of data or in writing the manuscript or the decision to publish.

***Financial support.*** This work was supported by the Erasmus University Medical Centre, Rotterdam, the Netherlands, MRace Grant (2019).

## References

[1] Milstone AM, Carroll KC, Ross T, Shangraw KA, Perl TM. Community-associated methicillin-resistant *Staphylococcus aureus* strains in pediatric intensive care unit. Emerg Infect Dis 2010; 16:647–55. 20350379 10.3201/eid1604.090107 PMC3321932

[2] Bottery MJ, Pitchford JW, Friman VP. Ecology and evolution of antimicrobial resistance in bacterial communities. Isme J 2021; 15:939–48. 33219299 10.1038/s41396-020-00832-7 PMC8115348

[3] Liu C, Bayer A, Cosgrove SE, et al Clinical practice guidelines by the Infectious Diseases Society of America for the treatment of methicillin-resistant *Staphylococcus aureus* infections in adults and children. Clin Infect Dis 2011; 52:e18–55. 21208910 10.1093/cid/ciq146

[4] Rybak MJ, Le J, Lodise TP, et al Therapeutic monitoring of vancomycin for serious methicillin-resistant *Staphylococcus aureus* infections: a revised consensus guideline and review by the American Society of Health-System Pharmacists, the Infectious Diseases Society of America, the Pediatric Infectious Diseases Society, and the Society of Infectious Diseases Pharmacists. Am J Health Syst Pharm 2020; 77:835–64. 32191793 10.1093/ajhp/zxaa036

[5] Fiorito TM, Luther MK, Dennehy PH, LaPlante KL, Matson KL. Nephrotoxicity with vancomycin in the pediatric population: a systematic review and meta-analysis. Pediatr Infect Dis J 2018; 37:654–61. 29280786 10.1097/INF.0000000000001882

[6] Hoang J, Dersch-Mills D, Bresee L, Kraft T, Vanderkooi OG. Achieving therapeutic vancomycin levels in pediatric patients. Can J Hosp Pharm 2014; 67:416–22. 25548398 10.4212/cjhp.v67i6.1403 PMC4275137

[7] Ter Heine R, Keizer RJ, van Steeg K, et al Prospective validation of a model-informed precision dosing tool for vancomycin in intensive care patients. Br J Clin Pharmacol 2020; 86:2497–506. 32415710 10.1111/bcp.14360 PMC7688533

[8] Chung E, Sen J, Patel P, Seto W. Population pharmacokinetic models of vancomycin in paediatric patients: a systematic review. Clin Pharmacokinet 2021; 60:985–1001. 34002357 10.1007/s40262-021-01027-9

[9] Cunio CB, Uster DW, Carland JE, et al Towards precision dosing of vancomycin in critically ill patients: an evaluation of the predictive performance of pharmacometric models in ICU patients. Clin Microbiol Infect. Published online 13 July 2020. doi:10.1016/j.cmi.2020.07.005 10.1016/j.cmi.2020.07.005 32673799

[10] Uster DW, Stocker SL, Carland JE, et al A model averaging/selection approach improves the predictive performance of model-informed precision dosing: vancomycin as a case study. Clin Pharmacol Ther 2021; 109:175–83. 32996120 10.1002/cpt.2065

[11] Boer DP, de Rijke YB, Hop WC, Cransberg K, Dorresteijn EM. Reference values for serum creatinine in children younger than 1 year of age. Pediatr Nephrol 2010; 25:2107–13. 20505955 10.1007/s00467-010-1533-y PMC2923720

[12] Schwartz GJ, Muñoz A, Schneider MF, et al New equations to estimate GFR in children with CKD. J Am Soc Nephrol 2009; 20:629–37. 19158356 10.1681/ASN.2008030287 PMC2653687

[13] Anderson BJ, Allegaert K, Van den Anker JN, Cossey V, Holford NH. Vancomycin pharmacokinetics in preterm neonates and the prediction of adult clearance. Br J Clin Pharmacol 2007; 63:75–84. 16869817 10.1111/j.1365-2125.2006.02725.x PMC2000709

[14] Capparelli EV, Lane JR, Romanowski GL, et al The influences of renal function and maturation on vancomycin elimination in newborns and infants. J Clin Pharmacol 2001; 41:927–34. 11549096 10.1177/00912700122010898

[15] Dao K, Guidi M, André P, et al Optimisation of vancomycin exposure in neonates based on the best level of evidence. Pharmacol Res 2020; 154:104278. 31108184 10.1016/j.phrs.2019.104278

[16] Frymoyer A, Hersh AL, El-Komy MH, et al Association between vancomycin trough concentration and area under the concentration-time curve in neonates. Antimicrob Agents Chemother 2014; 58:6454–61. 25136027 10.1128/AAC.03620-14 PMC4249374

[17] Jarugula P, Akcan-Arikan A, Munoz-Rivas F, Moffett BS, Ivaturi V, Rios D. Optimizing vancomycin dosing and monitoring in neonates and infants using population pharmacokinetic modeling. Antimicrob Agents Chemother 2022; 66:e0189921. 35293782 10.1128/aac.01899-21 PMC9046768

[18] Li ZL, Liu YX, Jiao Z, et al Population pharmacokinetics of vancomycin in Chinese ICU neonates: initial dosage recommendations. Front Pharmacol 2018; 9:603. 29997498 10.3389/fphar.2018.00603 PMC6029141

[19] Lo YL, van Hasselt JG, Heng SC, Lim CT, Lee TC, Charles BG. Population pharmacokinetics of vancomycin in premature Malaysian neonates: identification of predictors for dosing determination. Antimicrob Agents Chemother 2010; 54:2626–32. 20385872 10.1128/AAC.01370-09 PMC2876370

[20] Marqués-Miñana MR, Saadeddin A, Peris JE. Population pharmacokinetic analysis of vancomycin in neonates: a new proposal of initial dosage guideline. Br J Clin Pharmacol 2010; 70:713–20. 21039765 10.1111/j.1365-2125.2010.03736.x PMC2997311

[21] Tseng SH, Lim CP, Chen Q, Tang CC, Kong ST, Ho PC. Evaluating the relationship between vancomycin trough concentration and 24-hour area under the concentration-time curve in neonates. Antimicrob Agents Chemother 2018; 62:e01647-17. 29358290 10.1128/AAC.01647-17 PMC5914004

[22] Chen Y, Wu D, Dong M, et al Population pharmacokinetics of vancomycin and AUC-guided dosing in Chinese neonates and young infants. Eur J Clin Pharmacol 2018; 74:921–30. 29602981 10.1007/s00228-018-2454-0

[23] Colin PJ, Allegaert K, Thomson AH, et al Vancomycin pharmacokinetics throughout life: results from a pooled population analysis and evaluation of current dosing recommendations. Clin Pharmacokinet 2019; 58:767–80. 30656565 10.1007/s40262-018-0727-5

[24] Germovsek E, Osborne L, Gunaratnam F, et al Development and external evaluation of a population pharmacokinetic model for continuous and intermittent administration of vancomycin in neonates and infants using prospectively collected data. J Antimicrob Chemother 2019; 74:1003–11. 30668696 10.1093/jac/dky525

[25] Mehrotra N, Tang L, Phelps SJ, Meibohm B. Evaluation of vancomycin dosing regimens in preterm and term neonates using Monte Carlo simulations. Pharmacotherapy 2012; 32:408–19. 22488303 10.1002/j.1875-9114.2012.01029.x

[26] Hadi OA, Al Omar S, Nazer LH, Mubarak S, Le J. Vancomycin pharmacokinetics and predicted dosage requirements in pediatric cancer patients. J Oncol Pharm Pract 2016; 22:448–53. 26079639 10.1177/1078155215591386

[27] Chuphan C, Sukarnjanaset W, Puthanakit T, Wattanavijitkul T. Population pharmacokinetics and pharmacodynamics of vancomycin in pediatric patients with various degrees of renal function. J Pediatr Pharmacol Ther 2022; 27:419–27. 35845555 10.5863/1551-6776-27.5.419 PMC9268109

[28] De Cock RF, Allegaert K, Brussee JM, et al Simultaneous pharmacokinetic modeling of gentamicin, tobramycin and vancomycin clearance from neonates to adults: towards a semi-physiological function for maturation in glomerular filtration. Pharm Res 2014; 31:2643–54. 24789450 10.1007/s11095-014-1361-z PMC4749758

[29] Kloprogge F, Hill LF, Booth J, et al Revising pediatric vancomycin dosing accounting for nephrotoxicity in a pharmacokinetic-pharmacodynamic model. Antimicrob Agents Chemother 2019; 63:e00067-19. 30833429 10.1128/AAC.00067-19 PMC6496060

[30] Lanke S, Yu T, Rower JE, Balch AH, Korgenski EK, Sherwin CM. AUC-guided vancomycin dosing in adolescent patients with suspected sepsis. J Clin Pharmacol 2017; 57:77–84. 27291466 10.1002/jcph.782

[31] Le J, Bradley JS, Murray W, et al Improved vancomycin dosing in children using area under the curve exposure. Pediatr Infect Dis J 2013; 32:e155–63. 23340565 10.1097/INF.0b013e318286378e PMC3632448

[32] Lv CL, Lu JJ, Chen M, et al Vancomycin population pharmacokinetics and dosing recommendations in haematologic malignancy with augmented renal clearance children. J Clin Pharm Ther 2020; 45:1278–87. 32557716 10.1111/jcpt.13206

[33] Stockmann C, Sherwin CM, Zobell JT, et al Population pharmacokinetics of intermittent vancomycin in children with cystic fibrosis. Pharmacotherapy 2013; 33:1288–96. 23824677 10.1002/phar.1320

[34] Zhang T, Cheng H, Pan Z, et al Desired vancomycin trough concentration to achieve an AUC(0-24)/MIC ≥400 in Chinese children with complicated infectious diseases. Basic Clin Pharmacol Toxicol 2020; 126:75–85. 31403243 10.1111/bcpt.13303

[35] Zhao W, Zhang D, Fakhoury M, et al Population pharmacokinetics and dosing optimization of vancomycin in children with malignant hematological disease. Antimicrob Agents Chemother 2014; 58:3191–9. 24663023 10.1128/AAC.02564-13 PMC4068451

[36] Alsultan A, Abouelkheir M, Alqahtani S, et al Optimizing vancomycin monitoring in pediatric patients. Pediatr Infect Dis J 2018; 37:880–5. 29461449 10.1097/INF.0000000000001943

[37] Avedissian SN, Bradley E, Zhang D, et al Augmented renal clearance using population-based pharmacokinetic modeling in critically ill pediatric patients. Pediatr Crit Care Med 2017; 18:e388–94. 28640009 10.1097/PCC.0000000000001228

[38] Rhodin MM, Anderson BJ, Peters AM, et al Human renal function maturation: a quantitative description using weight and postmenstrual age. Pediatr Nephrol 2009; 24:67–76. 18846389 10.1007/s00467-008-0997-5

[39] Ruggiero A, Ariano A, Triarico S, Capozza MA, Ferrara P, Attinà G. Neonatal pharmacology and clinical implications. Drugs Context 2019; 8:212608. 31692800 10.7573/dic.212608 PMC6821278

[40] Kishk OA, Lardieri AB, Heil EL, Morgan JA. Vancomycin AUC/MIC and corresponding troughs in a pediatric population. J Pediatr Pharmacol Ther 2017; 22:41–7. 28337080 10.5863/1551-6776-22.1.41 PMC5341531

[41] Issaranggoon Na Ayuthaya S, Katip W, Oberdorfer P, Lucksiri A. Correlation of the vancomycin 24-h area under the concentration-time curve (AUC(24)) and trough serum concentration in children with severe infection: a clinical pharmacokinetic study. Int J Infect Dis 2020; 92:151–9. 31935538 10.1016/j.ijid.2019.12.036

[42] Suchartlikitwong P, Anugulruengkitt S, Wacharachaisurapol N, et al Optimizing vancomycin use through 2-point AUC-based therapeutic drug monitoring in pediatric patients. J Clin Pharmacol 2019; 59:1597–605. 31342543 10.1002/jcph.1498

[43] Brandon HH, Burgess DS, Wallace KL, Autry EB, Olney KB. Vancomycin AUC(0-24) estimation using first-order pharmacokinetic methods in pediatric patients. Pharmacotherapy 2024; 44:294–300. 38533999 10.1002/phar.2916

[44] Hughes JH, Tong DMH, Faldasz JD, Frymoyer A, Keizer RJ. Evaluation of neonatal and paediatric vancomycin pharmacokinetic models and the impact of maturation and serum creatinine covariates in a large multicentre data set. Clin Pharmacokinet 2023; 62:67–76. 36404388 10.1007/s40262-022-01185-4 PMC9898357

[45] Hughes JH, Tong DMH, Lucas SS, Faldasz JD, Goswami S, Keizer RJ. Continuous learning in model-informed precision dosing: a case study in pediatric dosing of vancomycin. Clin Pharmacol Ther 2021; 109:233–42. 33068298 10.1002/cpt.2088 PMC7839485

[46] Alihodzic D, Broeker A, Baehr M, Kluge S, Langebrake C, Wicha SG. Impact of inaccurate documentation of sampling and infusion time in model-informed precision dosing. Front Pharmacol 2020; 11:172. 32194411 10.3389/fphar.2020.00172 PMC7063976

