# Supplementary material for: Prediction of Vancomycin Area Under the Curve With Trough Concentrations Only: Performance Evaluation of Pediatric Population Pharmacokinetic Models
Source: J Infect Dis. 2025 Feb 4;231(5):e882–90. doi: 10.1093/infdis/jiaf059 (PMC12128073; doi:10.1093/infdis/jiaf059)
Supplement: jiaf059_Supplementary_Data [file jiaf059_supplementary_data.docx]

**Supplementary material**

**Prediction of Vancomycin Area-under-the-curve using Trough Concentrations Only: Performance Evaluation of Pediatric Population Pharmacokinetic Models**

**Short title: Trough-based Vancomycin AUC_24h_ estimation**

Stef Schouwenburg (PharmD)^1,2^, Tim Preijers (PhD)^1.2^, Robert B. Flint (PhD)^1,2,3^, Enno D Wildschut^4^, Birgit C.P. Koch (PhD)^1,2,5^, Brenda C.M. de Winter (PhD)^1,2,5^, Alan Abdulla (PhD)^1,2^

^1^Department of Hospital Pharmacy, Erasmus University Medical Centre, Rotterdam, the Netherlands

^2^Rotterdam Clinical Pharmacometrics Group, Erasmus University Medical Centre, Rotterdam, the Netherlands

^3^Department of Neonatal and Pediatric Intensive Care, Division of Neonatology, Erasmus University Medical Centre-Sophia Children’s Hospital, Rotterdam, The Netherlands

^4^Department of Neonatal and Pediatric Intensive Care, Division of Pediatric Intensive Care, Erasmus University Medical Centre-Sophia Children’s Hospital, Rotterdam, The Netherlands

^5^Center for Antimicrobial Treatment Optimization Rotterdam (CATOR), Rotterdam, The Netherlands

Corresponding author: Dr. Alan Abdulla

Department of Hospital Pharmacy, Erasmus University Medical Centre, Rotterdam, the Netherlands

Postal Box 2040

3000 CA Rotterdam, The Netherlands

Tel.: +3110 7033202

Email: a.abdulla@erasmusmc.nl

**Additional modelling information**

Patient demographic and clinical data were collected retrospectively from patients admitted to Sophia Children’s Hospital Erasmus Medical Centre. Patients with both peak and trough (pre-dose) samples collected within the same day (24 hours) were identified. Peak concentrations were included even if sampled beyond 1 hour after vancomycin administration, provided that an elimination phase could be estimated between the collected peak and trough samples. This required a time interval of several hours between the trough and peak samples. Trough samples were verified using electronic patient records and were included if they were explicitly labeled as such.

Selected population pharmacokinetic models were encoded in NONMEM^®^ v7.4 (Icon Development Solutions, Hanover, MD, USA). Model evaluation (MAXEVAL = 0) was performed by introducing datasets with either a peak or trough sample, both samples, or no samples (*a priori*). Patients were extrapolated to predict steady-state concentrations (C_SS_). An ADDL and II variable were introduced and set to ‘720’ times an infusion rate of ‘6 hours’, resembling 180 days since start of treatment.

**Model selection**

Population pharmacokinetic models not suitable for evaluation were excluded based on the following criteria. Models were excluded when covariates were unavailable in the dataset (n=17) (1-16), model encoding from literature was not feasible and authors did not reply to inquiries (n=6) (17-21), small sample size (n=5) (22-26), models were published before the year 2000 (n=8) (27-34), or when the model was developed for continuous infusion (n=2) (35, 36).

| Author | Year | Sample size | Vancomycin dosage regimen | Total samples | Age, mean (SD) or as described | Female (%) | Weight (kg), mean (SD) or as described | SCr, µmol/L, mean (SD) or as described | eGFR, mL/min/1.73m2, mean (SD) or as described and estimation method |
| --- | --- | --- | --- | --- | --- | --- | --- | --- | --- |
| Anderson (37) | 2007 | 214 | 15 mg/kg/dose q12-24h | 604 (≥ 2) | Median: 11.9 days, range: 1–27 days | NA | Median: 1.3, range: 0.42–2.6 | NA | NA |
| Capparelli (38) | 2001 | 374 | 15 mg/kg/dose q12-24h | 1103 | Median: 27 days, IQR: 15–74 | 54 | 2.82 (SD: 2.33) | 0.7 (SD: 0.5 mg/dl) | NA |
| Chen (39) | 2018 | 213 | 10 or 15 mg/kg/dose (for bacteremia or meningitis respectively) q8-12h based on PMA and PNA in Neofax | 330 (≥ 1) | median: 26 days, range: 6–59 days | NA | median 2.73, range: 0.88–5.1 | median: 24.8, range: 9.7-63.6 | median: 68 (Range: 17–219), Old Schwartz equation 1984 for full-term infants (0.45 when SCr is in mg/dL) |
| Colin (40) | 2019 | 2554 | NA | 8300 | 13 datasets: Refer to original paper | 13 datasets: Refer to original paper | 13 datasets: Refer to original paper | 13 datasets: Refer to original paper | NA |
| Dao (41) | 2020 | 405 | Most frequent: 10-15 mg/kg/dose; median 13.7 mg/kg q6-48h | 1831 (≥ 1, mean 4.5) | median: 12.3 days, range: 0-146 days | NA | median: 1.1, range: 0.462-5.66 | median: 54, range: 5-276 | NA |
| Frymoyer (42) | 2014 | 249 | <88.4 𝜇mol/L: 20 mg/kg/dose q24h for < 2kg vs. 15 mg/kg/dose q12h ≥2 kg; ≥88.4 𝜇mol/L: 20 mg/kg followed by TDM | 1702 (≥ 1, mean 5) | median 19 days, IQR: 10-42 days | 48.6 | median 2.9, IQR: 1.6-3.7 | median 35.4, IQR: 26.5-53 | NA |
| Germovsek (43) | 2019 | 54 | Intermittent dosing based on BNF for Children: PMA <29 weeks: 15 mg/kg q24h; 29-35 weeks: q12h; >35 weeks: q8h; continuous dosing: 15 mg/kg LD followed by: 1) SCr<64 mmol/L: 30 mg/kg/day; 64-100 mmol/L: 25 mg/kg/day; >100-150 mmol/L: 15 mg/kg/day) | 183 (≥ 1) | median: 30 days, range: 1-156 days | NA | NA | median: 31, range: 18-98 | NA |
| Jarugula (44) | 2022 | 934 | 15 mg/kg/dose q24h | 2471 (≥ 1) | 57.9 days, IQR: 18.6 – 86.2 | 41.4 | 3.5, IQR: 1.97 – 4.91, range: 0.37 – 11.88 | 0.46 mg/dL, IQR: 0.25 – 0.51, range: 0.1 – 4.54 | NA |
| Li (45) | 2018 | 80 | Based on local protocol, 10-15 mg/kg/dose q8-12h | 165 (1 or 2) | NA | 32.5 | 2.87 (SD: 0.89) | 23.2 (SD: 10.4) | NA |
| Lo (46) | 2010 | 116 | NA | 835 (NA) | 6.61 days (SD: 5.07) | 43.1 | 0.963 (SD: 0.2552) | 76 (SD: 20) | NA |
| Marques-Minana (47) | 2020 | 70 | 15 mg/kg/dose q8-24h based on weight and PNA | NA | 16.9 days (SD: 10.9) | NA | 1.7 (SD: 0.8) | NA | NA |
| Mehrotra (48) | 2012 | 134 | Protocol based on GA, PNA and weight | 267 (2) | 26.8 days (SD: 24.3) | 46.3 | 2.5 (SD: 1.1) | 53.04 (SD: 33.59) | NA |
| Tseng (49) | 2018 | 76 | 10-15 mg/kg/dose q6-24h | 429 (≥ 1) | median: 17.4 days, IQR: 11.4-40.6 days, range: 4-223.7 days | 42.1 | median: 1.043, IQR: 0.811-1.919, range: 0.32-6.59 | NA | NA |

**Table S1:** Characteristics of included population pharmacokinetic models for cohort A (PNA<50 days). SD: Standard deviation; kg: kilogram; SCr: serum creatinine; CrCl: creatinine clearance; mg: milligram; q8h: every 8 hours; q12h: every 12 hours; NA: not applicable; q6h: every 6 hours; q24h: every 24 hours; TDM: therapeutic drug monitoring; IQR: interquartile; LD: loading dose; PNA: postnatal age; GA: gestational age; dl: deciliter.

| Author | No. of compartments | Model for IIV | IIV of CL | IIV of Vd | Error model | Significant covariates included | Model Evaluation Method |
| --- | --- | --- | --- | --- | --- | --- | --- |
| Anderson (37) | 1 | Exponential | 45.35% | 46.27% | Proportional and additive | Weight, ventilation, inotropics, PMA, CrCl | Internal (GOF, bootstrap) |
| Capparelli (38) | 1 | Exponential | 32% | 16.01% | Proportional | Weight, PNA, GA, SCr | Internal (GOF) |
| Chen (39) | 1 | Exponential | 26.80% | NA | Proportional and additive | Weight, PMA, SCr | Internal (GOF, NPDE, non-parametric bootstrap) / External |
| Colin (40) | 1 | Exponential | 27.91% | 27.3 | Proportional and additive | Weight, PMA, SCr | Internal (GOF, VPC, bootstrap) |
| Dao (41) | 1 | NA | 22.60% | NA | Exponential and additive | Weight, PMA, SCr | Internal (Bootstrap, GOF, VPC, NPDE) / External |
| Frymoyer (42) | 1 | Exponential | 21.60% | 10.90% | Proportional and additive | Weight, PMA, SCr | Internal (GOF, bootstrap, NPDE) |
| Germovsek (43) | 1 | Proportional | 32.97% | 32.41% | Proportional | Weight, PMA | Internal (GOF, VPC) / External |
| Jarugula (44) | 1 | Exponential | 22.99% | 24.99% | Proportional and additive | Weight, PMA, SCr | Internal (GOF, VPC, bootstrap) |
| Li (45) | 1 | Exponential | 37.90% | NA | Proportional | Weight, SCr | Internal (GOF, NPDE, nonparametric bootstrap) |
| Lo (46) | 1 | Exponential | 20.50% | 12.60% | Proportional and additive | PMA, SGA, weight | Internal (GOF, bootstrap) |
| Marques-Minana (47) | 1 | Additive | 35.60% | 19.30% | Additive | PMA, amoxicillin-clavulanic acid, spironolactone | Internal (GOF, bootstrap, VPC) / External |
| Mehrotra (48) | 1 | Exponential | 25.30% | 21.80% | Proportional and additive | PMA, weight, SCr | Internal (GOF) |
| Tseng (49) | 1 | Exponential | 25.40% | 9.60% | Proportional and additive | Weight, PMA, SCr | Internal (GOF, VPC, bootstrap) |

**Table S2:** Properties and structure of the evaluated population pharmacokinetic models for cohort A (PNA<50 days). No. of compartments: number of compartments; IIV: interindividual variability; RV: residual variability; NA: not applicable; PMA: postmenstrual age; SCr: Serum creatinine; GOF: goodness of fit; NPDE: normalized prediction distribution errors; VPC: visual predictive check; SGA: small for gestational age; CrCl: creatinine clearance; PNA: postnatal age; GA: gestational age.

| Author | Year | Sample size | Vancomycin dosage regimen | Total samples | Age, mean (SD) or as described | Female (%) | Weight (kg), mean (SD) or as described | SCr, µmol/L, mean (SD) or as described | eGFR, mL/min/1.73m2, mean (SD) or as described and estimation method |
| --- | --- | --- | --- | --- | --- | --- | --- | --- | --- |
| Abdel Hadi (50) | 2016 | 49 | Mean 10.5 mg/kg/dose q6-8h | 120 (≥ 2) | 6 years (SD: 2.46) | 44.9 | 19.6 (SD: 6.95) | 35.89 (SD: 10.43) | - |
| Alsultan (51) | 2018 | 76 | 15 mg/kg/dose q6h (average 61.3 mg/kg/day) | 122 (2) | 5.8 years (SD: 2.9) | 39.5 | 18.1 (SD: 8.5) | 33.592 (SD: 10.61) | - |
| Avedissian (52) | 2017 | 250 | median: 45 mg/kg/day (IQR 39.97-58.61) or 16.63 mg/kg/dose (IQR 12.81-16.16) | 658 (NA) | median: 9.8 years, range: 3.2-14 years | 91.2 | median: 30, range: 15-50 | median: 35.4, range: 26.5-47.7 | - |
| Chuphan (53) | 2022 | 212 | Median 54.2 mg/kg/day, range 9.7 – 131.1 mg/kg/day, q6-24h | 348 (≥ 1) | Median: 3.5 years, IQR: 0.9 – 10.9 | 56.3 | Median: 14, IQR: 7.2 – 30.4 | Median 0.38 mg/dL, IQR: 0.25 – 0.59 | Median 108.9, IQR: 70.9 – 151.3, Schwartz |
| de Cock (54) | 2014 | 429 | - | 1168 (NA) | median 16 days, range: 1 day to 17 years | - | median 1.8, range: 0.415-85 | median 51, range: 7-144.1 | - |
| Kloprogge (55) | 2019 | 616 | NA | 4137 (≥ 2) | 61 months, range: 0.03-255 months | - | 19, range: 0.742-95 | 39, range: 5-892 | - |
| Lanke (56) | 2017 | 463 | 15-20 mg/kg/dose q6-12h | 1107 (mean 2.4) | median: 15.6 years, IQR: 14-17.5 years | 42.5 | median: 58.9, IQR: 45.8-72.2 | median: 54.8, IQR: 44.2-69.8 | NA (SD: NA), median CrCl: 108.1, IQR:88.5-133, modified Schwartz equation |
| Le (57) | 2013 | 702 | mean 45 ± 12 mg/kg/day divided q6h or q8h | 1660 (≥ 1) | median 6.6 years, IQR: 2.2-13.4 years | 47.2 | median: 22.8, IQR: 12.6-46 | 42.43 (SD: 29.17) | - |
| Lv (58) | 2020 | 53 | 7-21 mg/kg/dose q6-12h | 106 (≥ 1) | 8.62 years (SD: 4.13), range: 2.24-17.87 years | 58.5 | 28.12 (SD: 14.67), range: 11-72 | 29.17 mg/dL (SD: 13.26), range: 4.95-61.88 | 258.08 (SD: 165.4), <17 years old: Modified Schwartz (with k=40 in infants <1 , 48.6 for children <13 /adolescent girls and 61.88 in adolescent boys, and SCr in mmol/L); 17-18 old: Cockcroft-Gault |
| Stockmann (59) | 2014 | 67 | mean dose 16.5 ± 4.2 mg/kg/dose q6-12h | 486 (mean 7.9 ± 9.6 at Cardinal Glennon; 6.3 ± 6.8 at Intermountain Primary Children's Medical Center) | median 13.9 years, IQR: 8-17 years | 59.7 | median 41.2, IQR: 25.5-56.8 | - | - |
| Zhang (60) | 2020 | 201 | median 40 mg/kg/day (IQR: 29-40 mg/kg/day) | 383 (≥ 1) | median: 2.5 years, IQR: 0.7-6.1 years | 39.3 | median: 13, range: 0.8-20.8 | median: 25, IQR: 19-32.5 | median: 80.5 (IQR: 55.7-125.5), Schwartz |
| Zhao (61) | 2014 | 70 | 40-60 mg/kg/day divided q6h | 98 (NA) | 6.8 years (SD: 4.8), median: 5.6 years, range: 0.3-17.7 years | 41.4 | 25.7 (SD: 5.5), median: 20.2, range: 5.6-71 | 32 (SD: 17), median: 30, range: 10-141 | 115.49 (SD: 36.59), median 191, range: 48.7-457, Schwartz |

**Table S3:** Characteristics of included population pharmacokinetic models for cohort B (PNA≥50 days). SD: Standard deviation; kg: kilogram; SCr: serum creatinine; CrCl: creatinine clearance; q6h: every 6 hours; q8h: every 8 hours; mg: milligram; IQR: interquartile range; q12h: every 12 hours; NA; not applicable; q24h: every 24 hours.

| Author | No. of compartments | Model for IIV | IIV of CL | IIV of Vd | Error model | Significant covariates included | Model Evaluation Method |
| --- | --- | --- | --- | --- | --- | --- | --- |
| Abdel Hadi (50) | 1 | - | 33.94% | - | Additive | Weight | Internal (bootstrapping, GOF) |
| Alsultan (51) | 1 | Proportional | 15% | 11.60% | Proportional | Weight | Internal (OFV, physiologic plausibility, SE of parameter estimates and GOF) / external |
| Avedissian (52) | 1 | - | 38.70% | 34.90% | Additive | Weight, SCr | Internal (bootstrap, scatter plots) |
| Chuphan (53) | 1 | Exponential | 34.8% | 39.6% | Proportional and additive | Weight, CrCl | Internal (GOF, VPC, bootstrap) |
| de Cock (54) | 2 | Exponential | 43.18% | - | Proportional and additive | Weight | Internal (GOF, VPC, NPDE, bootstrap) |
| Kloprogge (55) | 2 | Exponential | 50.4 | 232 | Additive | Weight, PMA, age-standardized serum creatinine (gender) | Internal (OFV, NPDE, qq plot, VPC) / External |
| Lanke (56) | 1 | - | 27.90% | 24.90% | Proportional and additive | Weight, CrCl | Internal (bootstrap, diagnostic plots, nested models evaluated using likelihood ratio test and non-nested models assessed by AIC, VPC) |
| Le (57) | 1 | - | 35% | 18% | Proportional | Weight, SCr, age | Internal (GOF, bootstrap) |
| Lv (58) | 1 | Exponential | 21.61% | - | Exponential | Weight | Internal (GOF, bootstrap, VPC, NPDE) |
| Stockmann (59) | 1 | Exponential | 26.90% | 39.90% | Proportional and additive | Weight | Internal (GOF, unstable models were excluded, nonparametric bootstrap, VPC) |
| Zhang (60) | 1 | Exponential | 39.06% | 79.42% | Additive | Weight | Internal (bootstrap, VPC, GOF plots) |
| Zhao (61) | 1 | Exponential | 34.80% | 77% | Proportional and additive | Weight, CrCl | Internal (GOF, bootstrap, VPC, NPDE) / External |

**Table S4:** Properties and structure of the evaluated population pharmacokinetic models for cohort B (PNA≥50 days). No. of compartments: number of compartments; IIV: interindividual variability; Cl: clearance; Vd: distribution volume; RV: residual variability; GOF: goodness-of-fit; SE: standard error; SCr: serum creatinine; PMA: postmenstrual age; OFV; objective function value; NPDE: normalized prediction distribution errors; VPC: visual predictive check; CrCl: creatinine clearance; AIC: akaike Information Criterion.


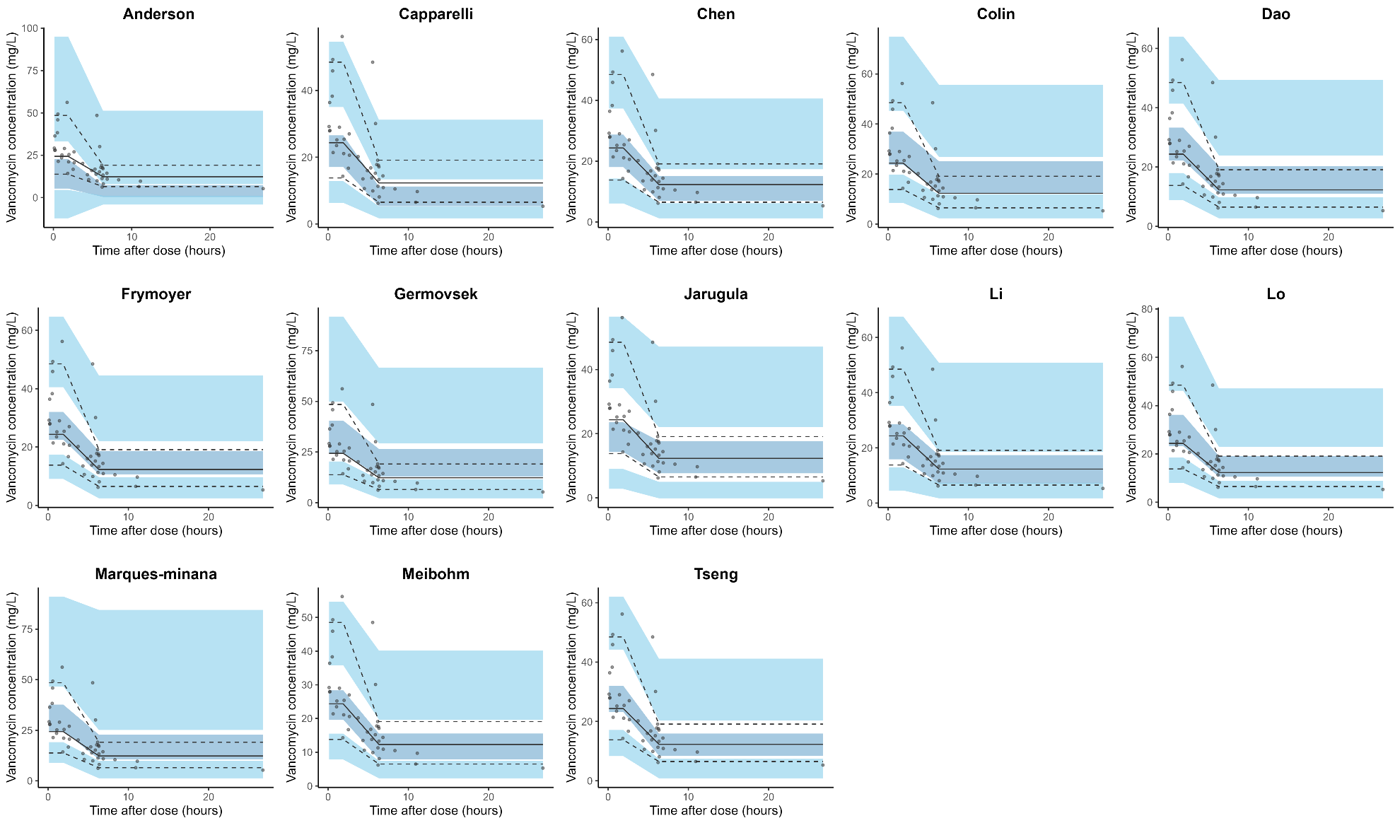


**Figure S1:** Visual predictive checks of the predicted vancomycin concentration-time profile in cohort A (PNA<50 days) for each of the evaluated 13 population pharmacokinetic models. Solid lines indicate the median of the data, dashed lines the 5th and 95th percentile of the data; the shaded areas indicate the 90% confidence intervals of the respective predictions obtained from the population pharmacokinetic models.


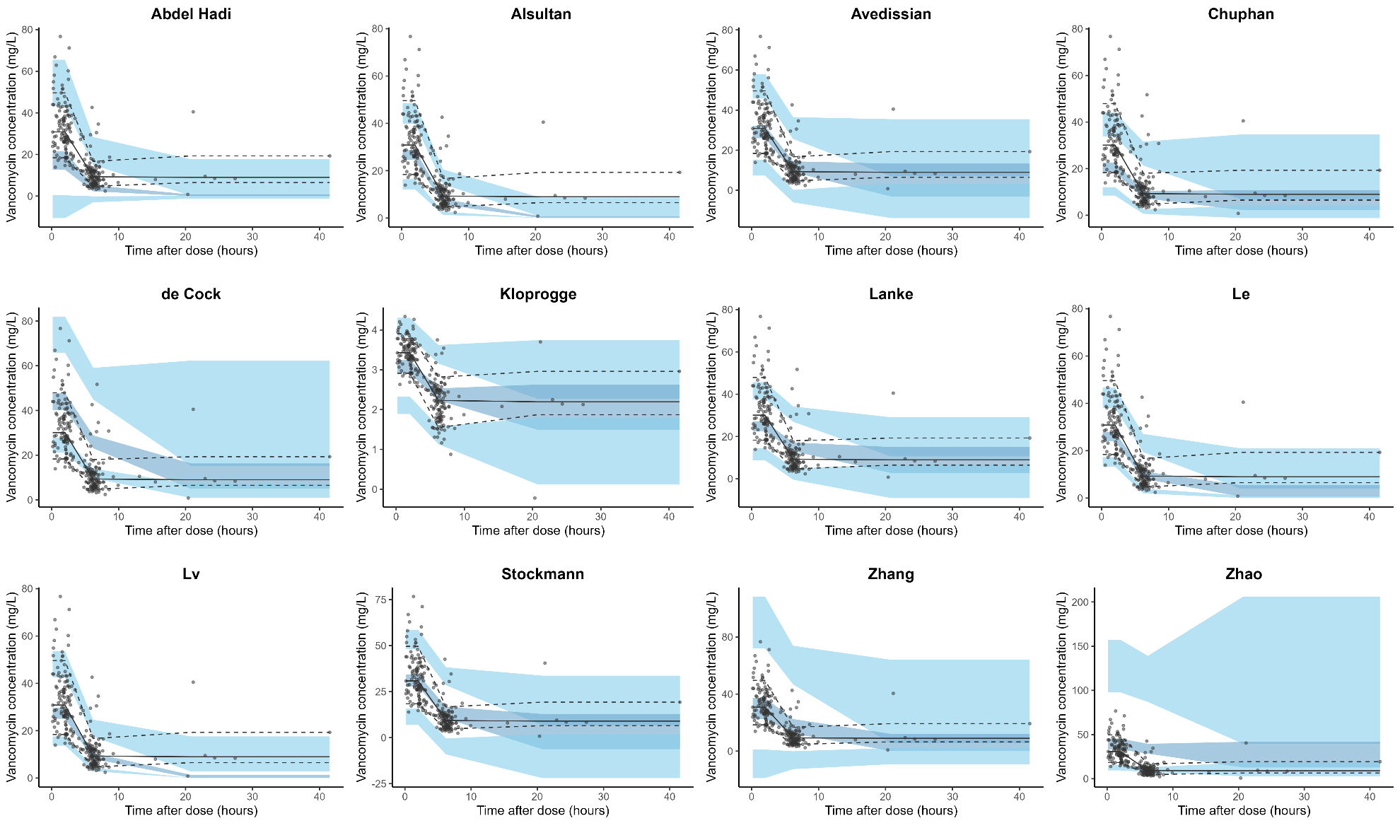


**Figure S2:** Visual predictive checks of the predicted vancomycin concentration-time profile in cohort B (PNA≥50 days) for each of the evaluated 12 population pharmacokinetic models. Solid lines indicate the median of the data, dashed lines the 5th and 95th percentile of the data; the shaded areas indicate the 90% confidence intervals of the respective predictions obtained from the population pharmacokinetic models.


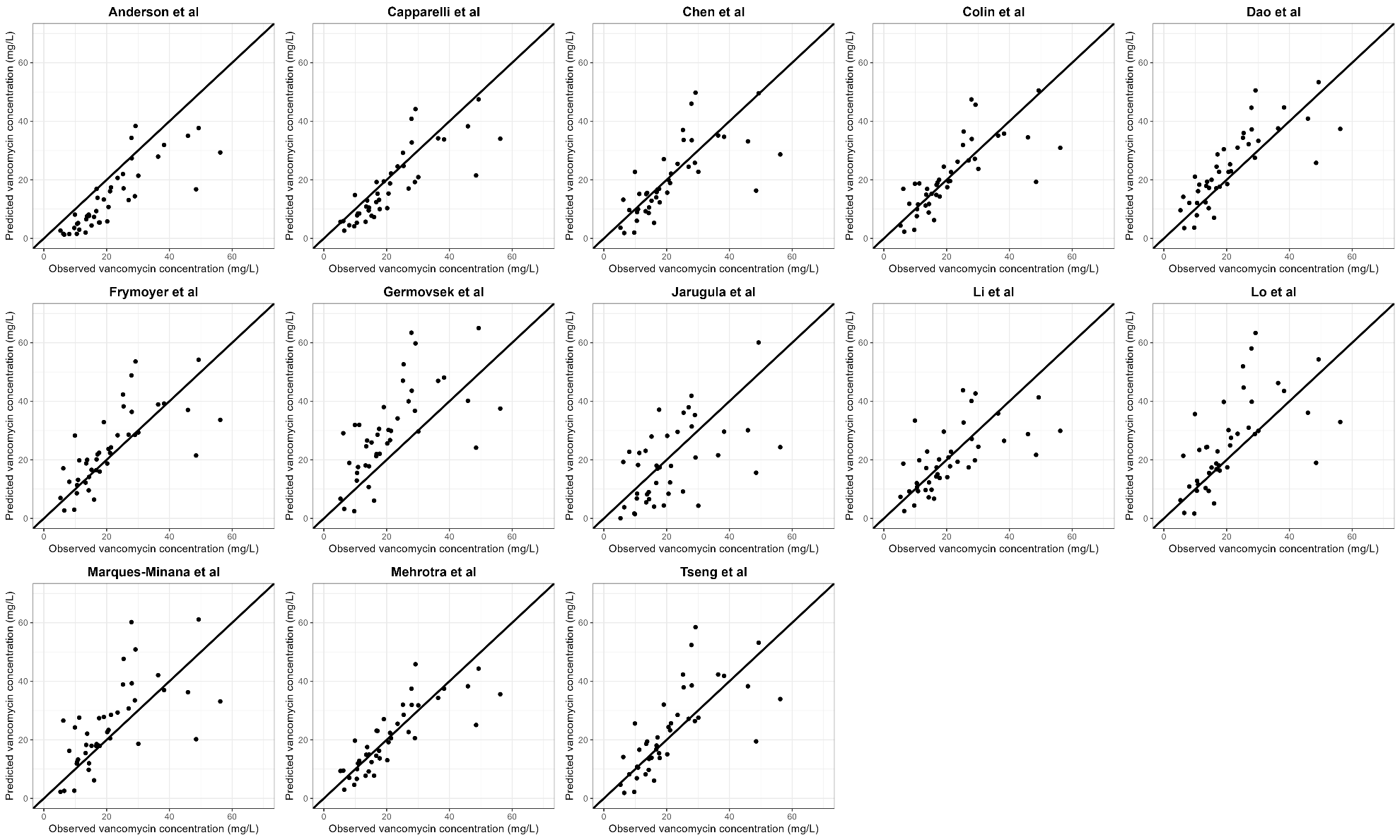


**Figure S3:** Goodness of fit plots showing observed versus predicted vancomycin concentrations by the 13 evaluated population pharmacokinetic models for cohort A (PNA<50 days).


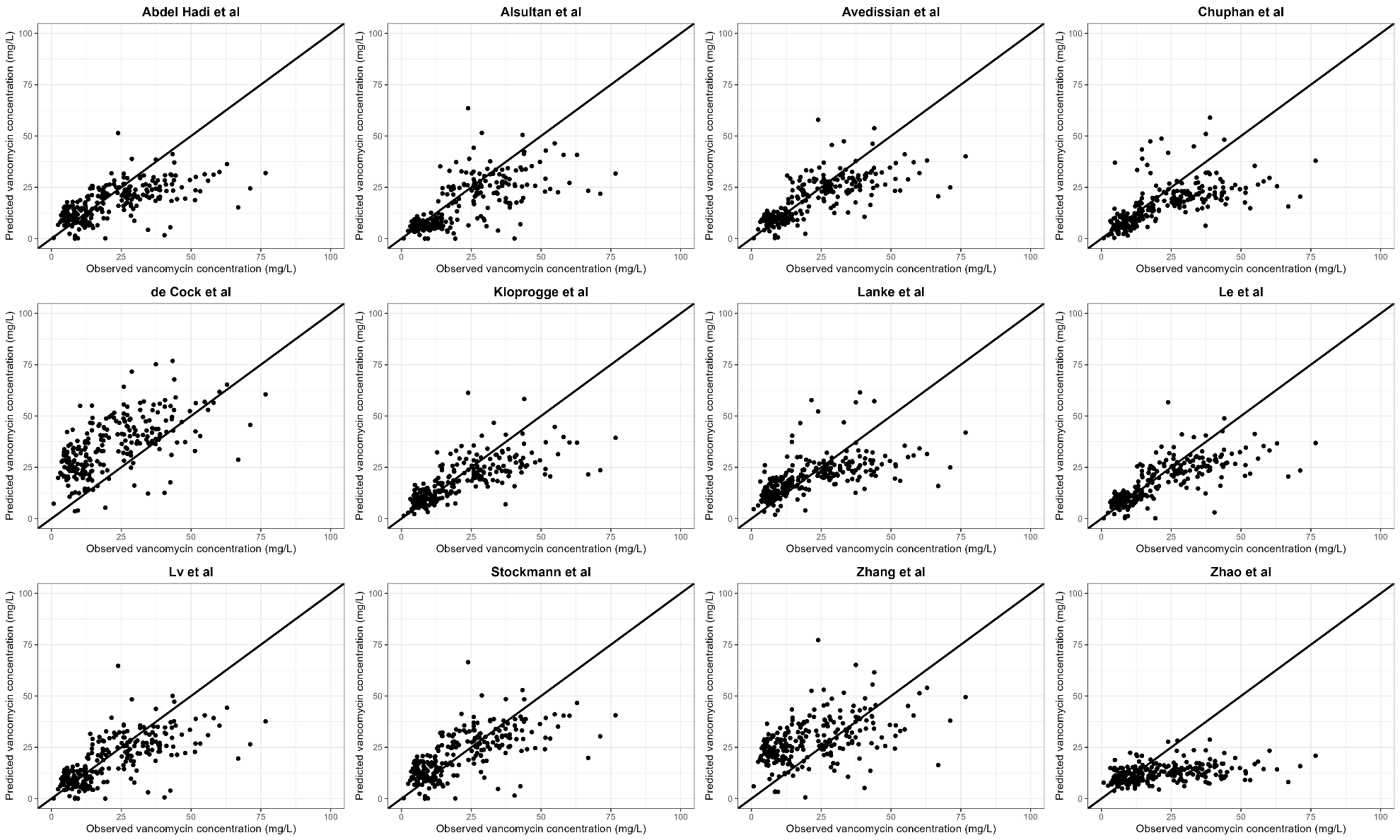


**Figure S4:** Goodness of fit plots illustrating observed versus predicted vancomycin concentrations by the 12 evaluated population pharmacokinetic models for cohort B (PNA≥50 days).


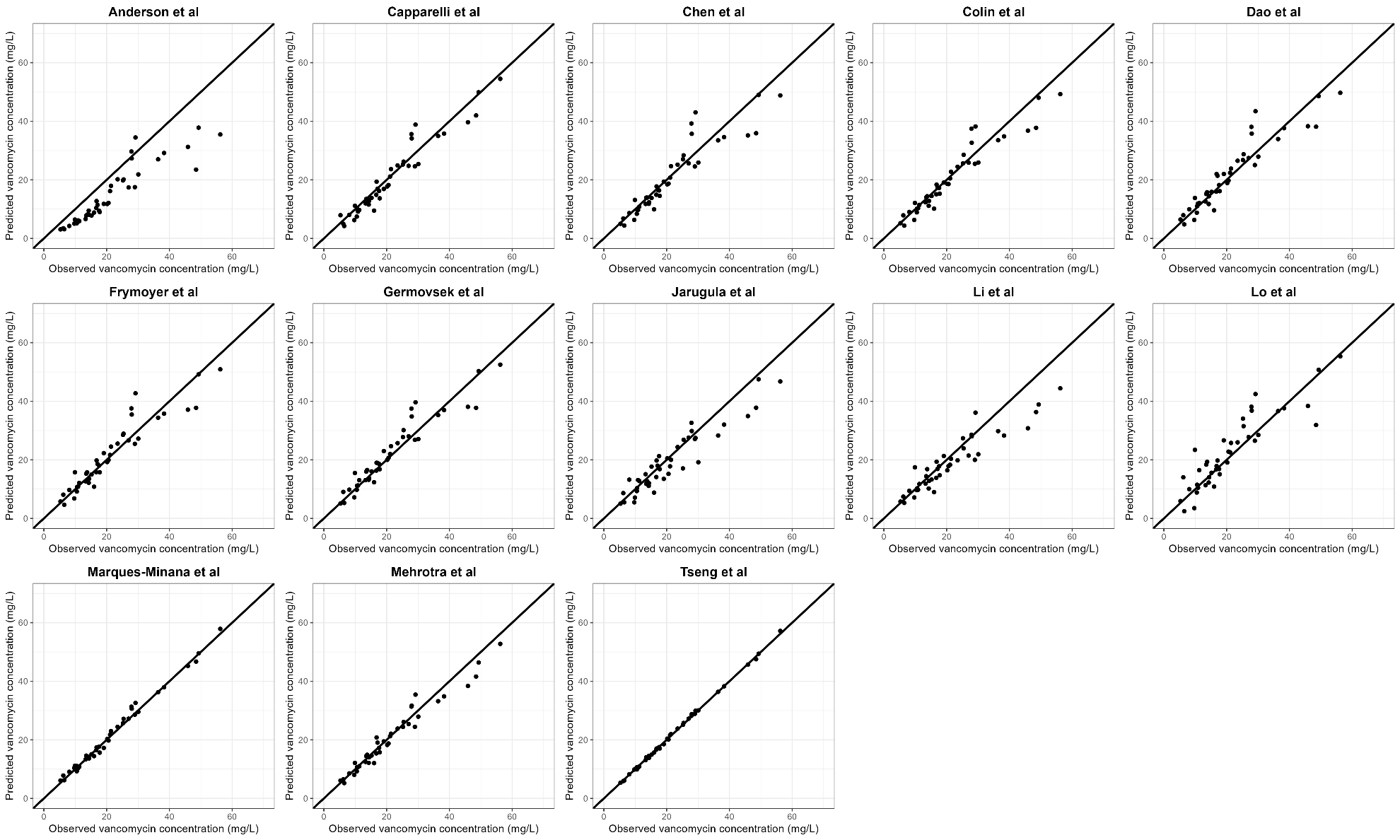


**Figure S5:** Goodness of fit plots showing observed versus individual predicted vancomycin concentrations by the 13 evaluated population pharmacokinetic models for cohort A (PNA<50 days).


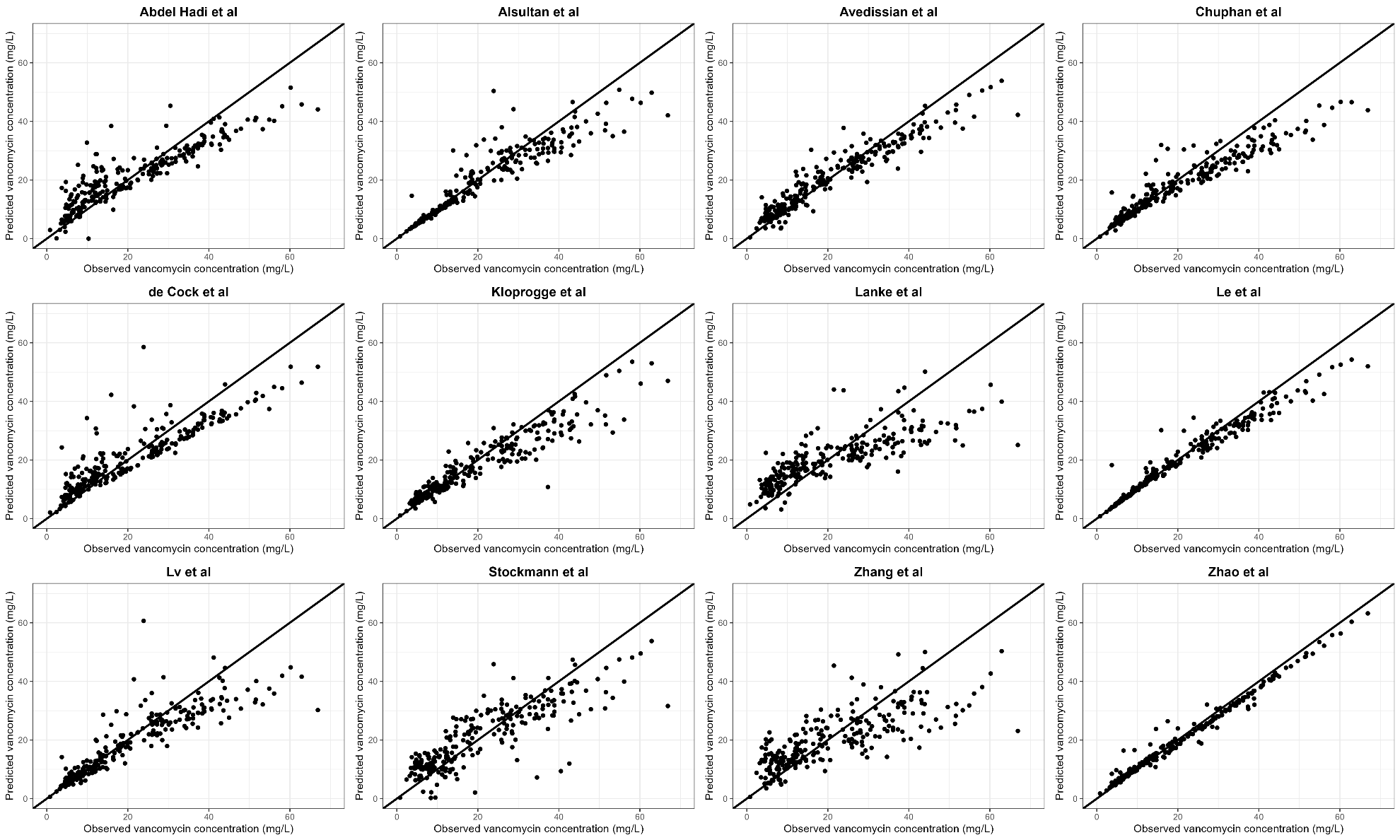


**Figure S6:** Goodness of fit plots showing observed versus individual predicted vancomycin concentrations by the 12 evaluated population pharmacokinetic models for cohort B (PNA≥50 days).

| Model | Scenario | rBias | Lower CI | Upper  CI | rRMSE |
| --- | --- | --- | --- | --- | --- |
| Anderson et al., 2007 | Peak + trough | -28.8% | -30.7% | -27.0% | 29.1% |
|  | Peak | -36.0% | -40.2% | -31.7% | 37.1% |
|  | Trough | -26.8% | -29.2% | -24.3% | 27.3% |
|  | *A priori* | -35.3% | -41.3% | -29.3% | 37.6% |
| Capparelli et al., 2001 | Peak + trough | -0.3% | -3.7% | 3.1% | 7.3% |
|  | Peak | -5.2% | -10.9% | 0.5% | 13.3% |
|  | Trough | -2.6% | -7.3% | 2.1% | 10.5% |
|  | *A priori* | -13.3% | -20.6% | -6.0% | 20.5% |
| Chen et al., 2018 | Peak + trough | -1.3% | -3.6% | 1.0% | 5.2% |
|  | Peak | -2.0% | -9.3% | 5.2% | 15.7% |
|  | Trough | -1.4% | -5.5% | 2.8% | 9.0% |
|  | *A priori* | -2.7% | -16.2% | 10.8% | 29.0% |
| Colin et al., 2019 | Peak + trough | -2.6% | -3.8% | -1.4% | 3.6% |
|  | Peak | -2.4% | -7.2% | 2.4% | 10.6% |
|  | Trough | -3.3% | -6.6% | -0.1% | 7.7% |
|  | *A priori* | -2.5% | -14.4% | 9.3% | 25.5% |
| Dao et al, 2020 | Peak + trough | 4.6% | 2.0% | 7.2% | 7.3% |
|  | Peak | 11.4% | 4.2% | 18.5% | 19.0% |
|  | Trough | 5.2% | 0.7% | 9.8% | 11.1% |
|  | *A priori* | 17.0% | 5.4% | 28.6% | 30.2% |
| Frymoyer et al., 2014 | Peak + trough | 4.1% | 1.4% | 6.7% | 6.9% |
|  | Peak | 9.8% | 1.2% | 18.5% | 21.0% |
|  | Trough | 4.9% | -0.4% | 10.2% | 12.4% |
|  | *A priori* | 15.2% | -0.4% | 30.8% | 36.8% |
| Germovsek et al., 2019 | Peak + trough | 6.9% | 4.5% | 9.4% | 8.7% |
|  | Peak | 21.6% | 10.4% | 32.8% | 32.3% |
|  | Trough | 13.0% | 7.2% | 18.8% | 17.9% |
|  | *A priori* | 43.7% | 21.7% | 65.6% | 64.2% |
| Jarugula et al., 2022 | Peak + trough | -7.2% | -13.4% | -0.9% | 15.1% |
|  | Peak | -3.4% | -16.8% | 10.1% | 29.1% |
|  | Trough | -13.8% | -24.5% | -3.2% | 26.7% |
|  | *A priori* | -9.3% | -31.9% | 13.3% | 49.3% |
| Li et al., 2018 | Peak + trough | -6.9% | -12.0% | -1.8% | 13.0% |
|  | Peak | 2.6% | -7.9% | 13.0% | 22.6% |
|  | Trough | -9.0% | -16.6% | -1.4% | 18.6% |
|  | *A priori* | 0.7% | -18.0% | 19.4% | 40.0% |
| Lo et al., 2010 | Peak + trough | 10.2% | 2.1% | 18.2% | 20.1% |
|  | Peak | 15.2% | 1.2% | 29.2% | 33.7% |
|  | Trough | 16.0% | 2.1% | 29.8% | 33.7% |
|  | *A priori* | 25.6% | 2.9% | 48.3% | 55.0% |
| Marques-Minana et al., 2020 | Peak + trough | 5.8% | 0.0% | 11.6% | 13.7% |
|  | Peak | 8.3% | -2.2% | 18.7% | 23.9% |
|  | Trough | 9.3% | 3.7% | 15.0% | 15.3% |
|  | *A priori* | 20.6% | 1.0% | 40.3% | 47.0% |
| Mehrotra et al., 2012 | Peak + trough | 0.3% | -2.3% | 2.9% | 5.6% |
|  | Peak | 2.5% | -2.6% | 7.5% | 11.1% |
|  | Trough | -2.2% | -5.0% | 0.6% | 6.4% |
|  | *A priori* | -0.6% | -10.3% | 9.0% | 20.7% |
| Tseng et al., 2018 | Peak + trough | 5.0% | -0.6% | 10.5% | 12.9% |
|  | Peak | -1.1% | -10.8% | 8.6% | 20.8% |
|  | Trough | 11.2% | 5.6% | 16.9% | 16.5% |
|  | *A priori* | 10.9% | -4.6% | 26.4% | 35.0% |

**Table S5:** The relative bias (rBias) with lower and upper confidence interval (CI) and relative root mean squared error (rRMSE) of the model-predicted AUC_24h_ versus the ‘true’ AUC_24h_ for all models for cohort A (PNA<50 days)).

| Model | Scenario | rBias | Lower CI | Upper  CI | rRMSE |
| --- | --- | --- | --- | --- | --- |
| Abdel Hadi et al., 2016 | Peak + trough | 1.4% | -1.7% | 4.4% | 17.3% |
|  | Peak | 21.0% | 15.2% | 26.8% | 38.8% |
|  | Trough | -19.7% | -22.0% | -17.5% | 23.5% |
|  | *A priori* | -11.1% | -15.5% | -6.6% | 27.4% |
| Alsultan et al., 2018 | Peak + trough | 3.5% | 2.0% | 5.0% | 9.1% |
|  | Peak | 0.5% | -1.8% | 2.8% | 13.0% |
|  | Trough | -0.1% | -2.9% | 2.7% | 15.6% |
|  | *A priori* | -8.3% | -12.8% | -3.8% | 26.9% |
| Avedissian et al., 2017 | Peak + trough | 2.4% | 1.4% | 3.3% | 5.8% |
|  | Peak | 5.8% | 4.2% | 7.5% | 11.0% |
|  | Trough | -5.0% | -7.7% | -2.3% | 16.1% |
|  | *A priori* | -3.5% | -7.3% | 0.2% | 21.4% |
| Chuphan et al., 2022 | Peak + trough | -7.3% | -9.9% | -4.7% | 16.3% |
|  | Peak | 1.5% | -2.4% | 5.5% | 22.3% |
|  | Trough | -21.5% | -24.8% | -18.2% | 28.6% |
|  | *A priori* | -18.7% | -24.9% | -12.5% | 39.6% |
| de Cock et al., 2014 | Peak + trough | 2.8% | -0.1% | 5.7% | 16.4% |
|  | Peak | 22.9% | 17.9% | 27.9% | 36.3% |
|  | Trough | -17.8% | -20.4% | -15.3% | 23.0% |
|  | *A priori* | 78.6% | 69.7% | 87.5% | 93.3% |
| Kloprogge et al., 2019 | Peak + trough | -3.5% | -5.2% | -1.8% | 10.1% |
|  | Peak | -0.1% | -2.5% | 2.2% | 13.4% |
|  | Trough | -10.5% | -12.8% | -8.1% | 17.0% |
|  | *A priori* | -7.0% | -10.8% | -3.1% | 22.8% |
| Lanke et al., 2017 | Peak + trough | -2.0% | -4.4% | 0.4% | 13.5% |
|  | Peak | -1.2% | -5.8% | 3.4% | 25.8% |
|  | Trough | -8.0% | -11.5% | -4.5% | 21.3% |
|  | *A priori* | -1.8% | -6.7% | 3.1% | 27.8% |
| Le et al., 2013 | Peak + trough | 2.8% | 1.2% | 4.3% | 9.2% |
|  | Peak | 12.1% | 8.0% | 16.2% | 26.3% |
|  | Trough | -7.4% | -9.7% | -5.0% | 15.2% |
|  | *A priori* | -6.9% | -10.3% | -3.5% | 20.4% |
| Lv et al., 2020 | Peak + trough | -1.4% | -3.1% | 0.2% | 9.4% |
|  | Peak | 1.7% | -0.9% | 4.3% | 14.8% |
|  | Trough | -4.2% | -6.5% | -1.8% | 14.0% |
|  | *A priori* | -2.6% | -7.5% | 2.3% | 27.6% |
| Stockmann et al., 2014 | Peak + trough | 8.0% | 4.6% | 11.4% | 20.9% |
|  | Peak | 10.9% | 7.0% | 14.8% | 24.4% |
|  | Trough | 6.7% | 2.0% | 11.4% | 27.6% |
|  | *A priori* | 10.5% | 5.0% | 16.1% | 32.9% |
| Zhang et al., 2020 | Peak + trough | 3.3% | -0.6% | 7.3% | 22.5% |
|  | Peak | 24.3% | 18.6% | 30.0% | 40.4% |
|  | Trough | 11.3% | 6.0% | 16.6% | 32.0% |
|  | *A priori* | 36.0% | 28.7% | 43.3% | 54.9% |
| Zhao et al., 2014 | Peak + trough | 7.1% | 2.5% | 11.6% | 26.7% |
|  | Peak | 36.8% | 30.5% | 43.0% | 50.9% |
|  | Trough | -35.6% | -41.9% | -29.3% | 50.4% |
|  | *A priori* | -30.3% | -36.6% | -24.0% | 46.7% |

**Table S6:** The relative bias (rBias) with lower and upper confidence interval (CI) and relative root mean squared error (rRMSE) of the model-predicted AUC_24h_ versus the ‘true’ AUC_24h_ for all models for cohort B (PNA≥50 days)).


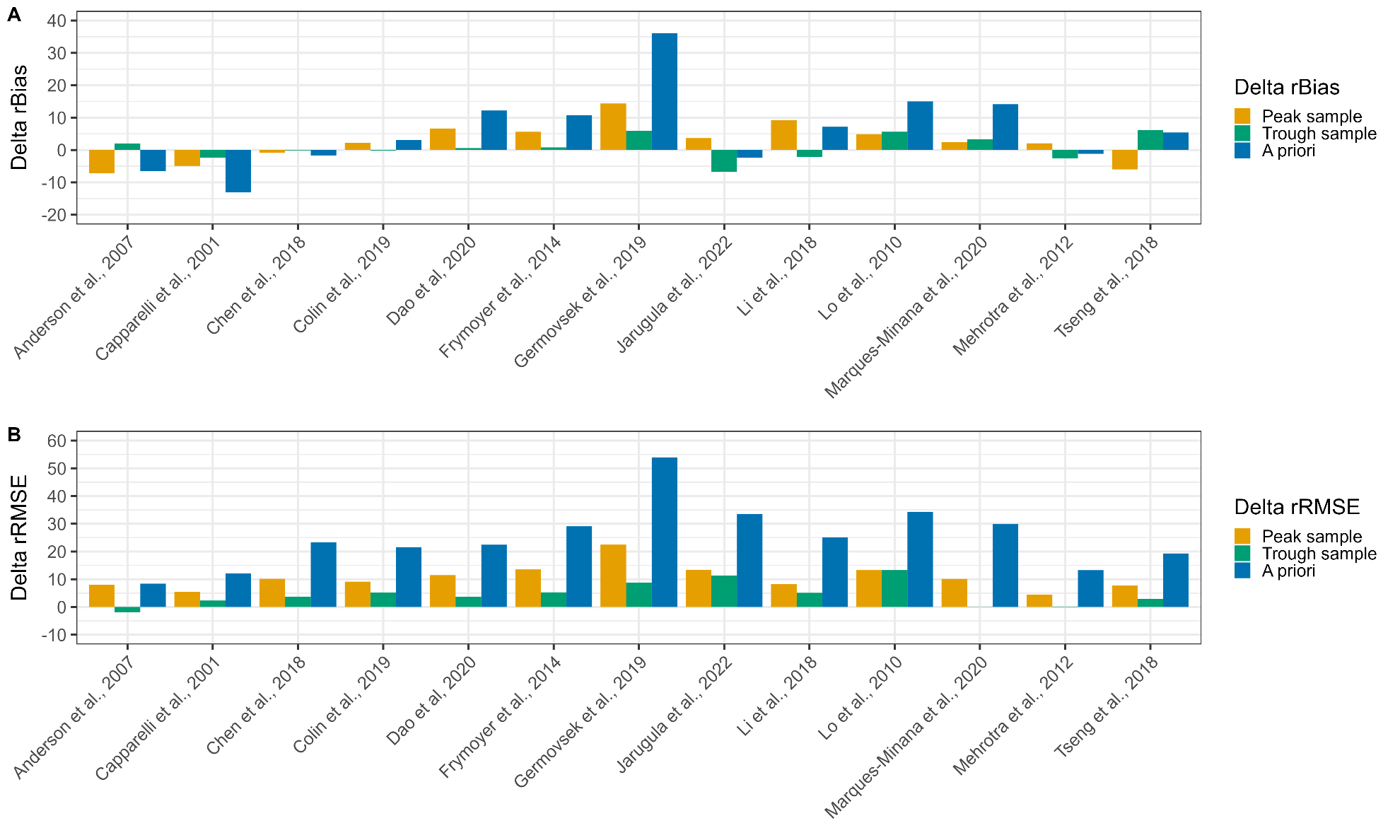


**Figure S7:** Delta relative bias (rBias) and relative root mean squared error (rRMSE) of the predicted AUC_24h_ versus the AUC_24h_ based on a peak and trough sample for cohort A (PNA<50 days). (a) peak sample; (b) trough sample; (c) providing covariate information solely (*a priori*).


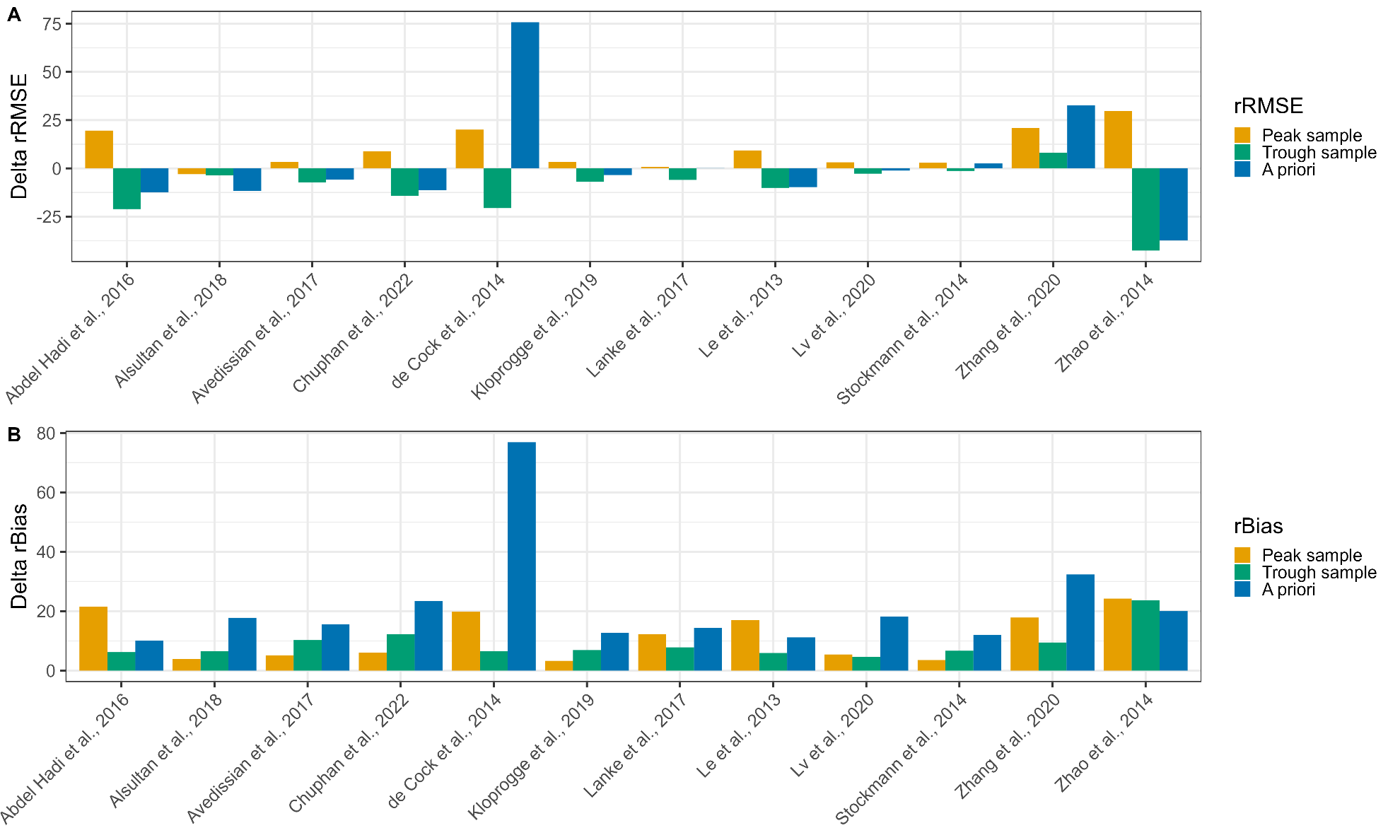


**Figure S8:** Delta relative bias (rBias) and relative root mean squared error (rRMSE) of the predicted AUC_24h_ versus the AUC_24h_ based on a peak and trough sample for cohort B (PNA≥50 days). (a) peak sample; (b) trough sample; (c) providing covariate information solely (*a priori*).

|  | **Models** | | | | | | | | | | | | |
| --- | --- | --- | --- | --- | --- | --- | --- | --- | --- | --- | --- | --- | --- |
| **Dosage advice** | **Anderson et al., 2007** | **Capparelli et al., 2001** | **Chen et al., 2018** | **Colin et al., 2019** | **Dao et al, 2020** | **Frymoyer et al., 2014** | **Germovsek et al., 2019** | **Jarugula et al., 2022** | **Li et al., 2018** | **Lo et al., 2010** | **Marques-Minana et al., 2020** | **Mehrotra et al., 2012** | **Tseng et al., 2018** |
| **No change** | 33.30% | 81.00% | 76.20% | 76.20% | 81.00% | 76.20% | 71.40% | 52.40% | 71.40% | 61.90% | 81.00% | 81.00% | 85.70% |
| **Dosage adjustment** | 66.70% | 19.00% | 23.80% | 23.80% | 19.00% | 23.80% | 28.60% | 47.60% | 28.60% | 38.10% | 19.00% | 19.00% | 14.30% |

**Table S7**: Percentage of subjects (cohort A, PNA<50 days) requiring a dosage adjustment based on the difference in ‘true’ AUC_24h_ and the trough-based model AUC_24h_.

|  | **Models** | | | | | | | | | | | |
| --- | --- | --- | --- | --- | --- | --- | --- | --- | --- | --- | --- | --- |
| **Dosage advice** | **Abdel Hadi et al., 2016** | **Alsultan et al., 2018** | **Avedissian et al., 2017** | **Chuphan et al., 2022** | **de Cock et al., 2014** | **Kloprogge et al., 2019** | **Lanke et al., 2017** | **Le et al., 2013** | **Lv et al., 2020** | **Stockmann et al., 2014** | **Zhang et al., 2020** | **Zhao et al., 2014** |
| **No change** | 51.60% | 73.80% | 69.80% | 47.60% | 51.60% | 73.80% | 70.60% | 74.60% | 77.00% | 50.80% | 52.40% | 26.20% |
| **Dosage adjustment** | 48.40% | 26.20% | 30.20% | 52.40% | 48.40% | 26.20% | 29.40% | 25.40% | 23.00% | 49.20% | 47.60% | 73.80% |

**Table S8:** Percentage of subjects (cohort B, PNA≥50 days) requiring a dosage adjustment based on the difference in ‘true’ AUC_24h_ and the trough-based model AUC_24h_.


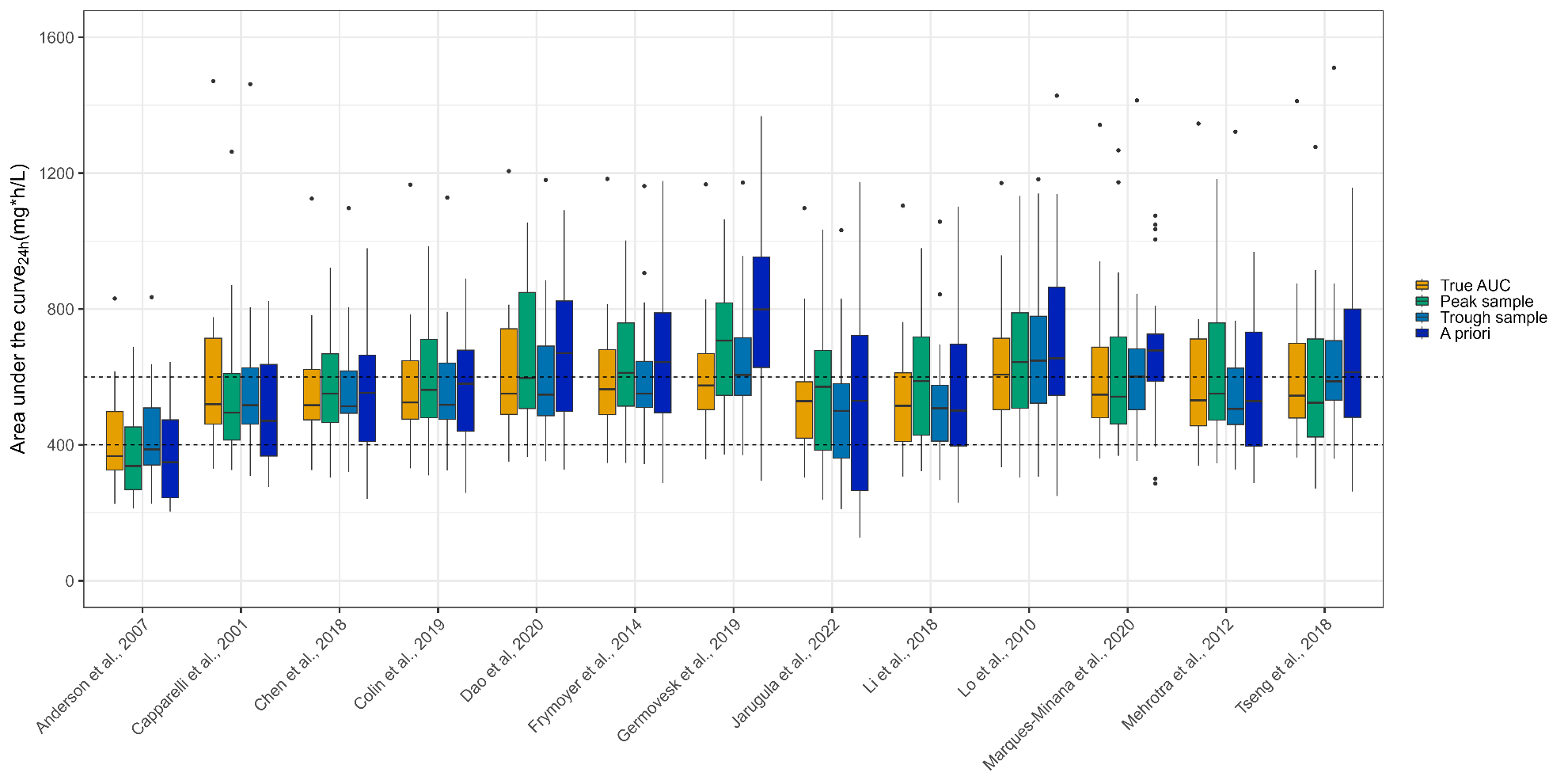


**Figure 5: MAP Bayesian analysis estimated and predicted vancomycin 24 hours area under the curve (AUC_24h_) for cohort A (PNA≤50 days). ‘**true’ AUC_24h_ (orange); a peak sample (green); a trough sample (blue); providing covariate information solely (a priori) (dark blue). Box plots represent the 25th, 50th and 75th percentiles. Whiskers cover the 1.5-fold interquartile range and outliers are marked as dots. The dashed line indicates the AUC_24h_/MIC target of 400 mg·h/L.

**
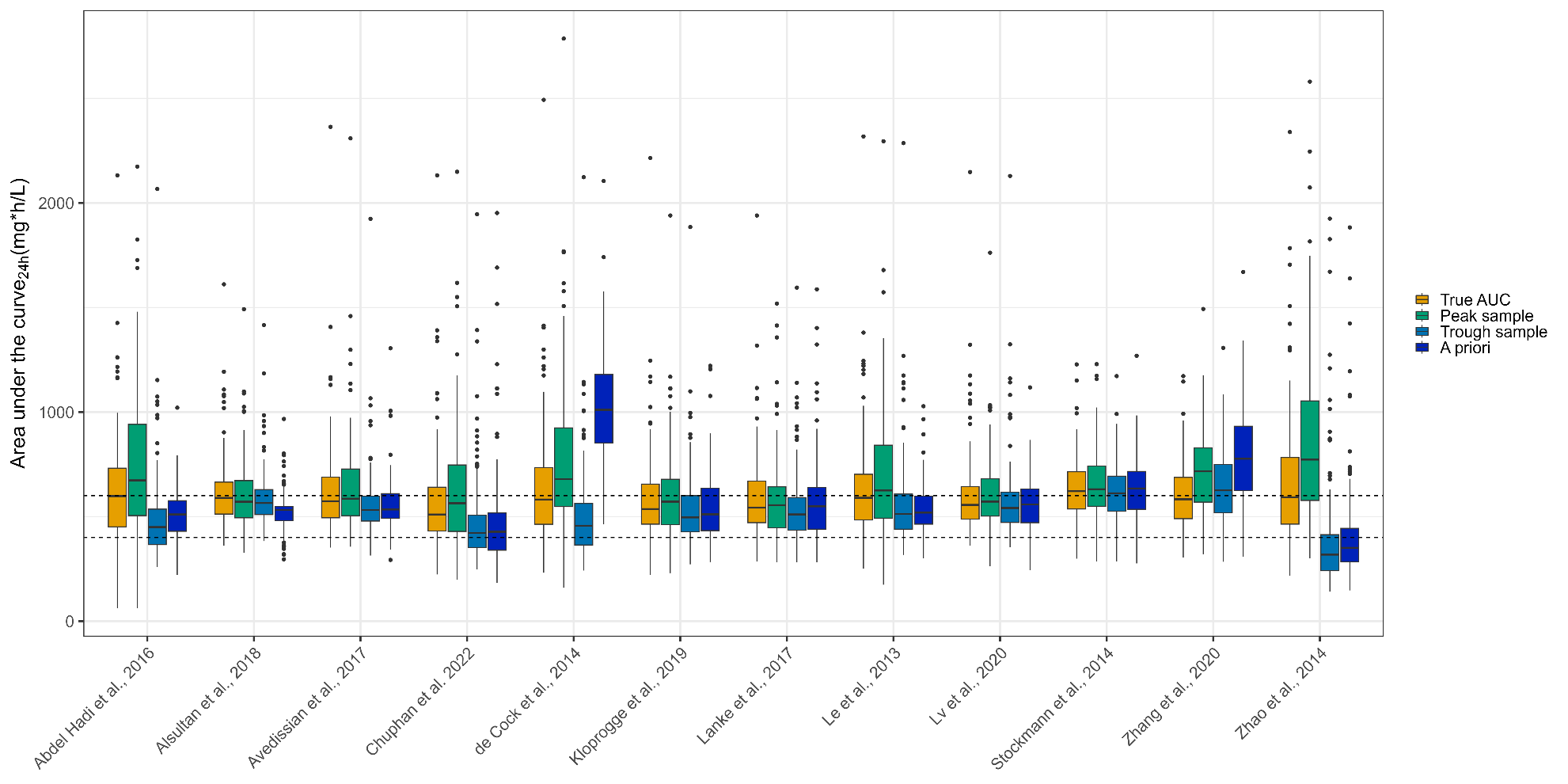
**

**Figure 6: MAP Bayesian analysis estimated and predicted vancomycin 24 hours area under the curve (AUC_24h_) for cohort B (PNA≥50 days).** ‘true’ AUC_24h_ (orange); a peak sample (green); a trough sample (blue); providing covariate information solely (a priori) (dark blue). Box plots represent the 25th, 50th and 75th percentiles. Whiskers cover the 1.5-fold interquartile range and outliers are marked as dots. The dashed line indicates the AUC_24h_/MIC target of 400 mg·h/L.

**References**

1. Song L, He CY, Yin NG, Liu F, Jia YT, Liu Y. A population pharmacokinetic model for individualised dosage regimens of vancomycin in Chinese neonates and young infants. Oncotarget. 2017;8(62):105211-21.

2. Reilly AM, Ding MX, Rower JE, Kiser TH. The Effectiveness of a Vancomycin Dosing Guideline in the Neonatal Intensive Care Unit for Achieving Goal Therapeutic Trough Concentrations. J Clin Pharmacol. 2019;59(7):997-1005.

3. Jacqz-Aigrain E, Leroux S, Thomson AH, Allegaert K, Capparelli EV, Biran V, et al. Population pharmacokinetic meta-analysis of individual data to design the first randomized efficacy trial of vancomycin in neonates and young infants. Journal of Antimicrobial Chemotherapy. 2019;74(8):2128-38.

4. Lu JJ, Chen M, Lv CL, Zhang R, Lu H, Cheng DH, et al. A Population Pharmacokinetics Model for Vancomycin Dosage Optimization Based on Serum Cystatin C. Eur J Drug Metab Pharmacokinet. 2020;45(4):535-46.

5. Moffett BS, Humlicek TJ, Akcan-Arikan A, Anders M, Tume S. Population Pharmacokinetics of Vancomycin in the Pediatric Ventricular Assist Device Population. Pediatr Crit Care Med. 2020;21(8):e566-e71.

6. Moffett BS, Ivaturi V, Morris J, Akcan Arikan A, Dutta A. Population Pharmacokinetic Assessment of Vancomycin Dosing in the Large Pediatric Patient. Antimicrob Agents Chemother. 2019;63(4).

7. Moffett BS, Morris J, Galati M, Munoz F, Arikan AA. Population Pharmacokinetics of Vancomycin in Pediatric Extracorporeal Membrane Oxygenation. Pediatr Crit Care Med. 2018;19(10):973-80.

8. Moffett BS, Morris J, Munoz F, Arikan AA. Population pharmacokinetic analysis of vancomycin in pediatric continuous renal replacement therapy. Eur J Clin Pharmacol. 2019;75(8):1089-97.

9. Back HM, Lee JB, Han N, Goo S, Jung E, Kim J, et al. Application of Size and Maturation Functions to Population Pharmacokinetic Modeling of Pediatric Patients. Pharmaceutics. 2019;11(6).

10. Cristea S, Allegaert K, Falcao AC, Falcao F, Silva R, Smits A, et al. Larger Dose Reductions of Vancomycin Required in Neonates with Patent Ductus Arteriosus Receiving Indomethacin versus Ibuprofen. Antimicrob Agents Chemother. 2019;63(8).

11. Zane NR, Reedy MD, Gastonguay MR, Himebauch AS, Ramsey EZ, Topjian AA, Zuppa AF. A Population Pharmacokinetic Analysis to Study the Effect of Therapeutic Hypothermia on Vancomycin Disposition in Children Resuscitated From Cardiac Arrest. Pediatr Crit Care Med. 2017;18(7):e290-e7.

12. Guilhaumou R, Marsot A, Dupouey J, Galambrun C, Boulamery A, Coze C, et al. Pediatric Patients With Solid or Hematological Tumor Disease: Vancomycin Population Pharmacokinetics and Dosage Optimization. Ther Drug Monit. 2016;38(5):559-66.

13. Bhongsatiern J, Stockmann C, Roberts JK, Yu T, Korgenski KE, Spigarelli MG, et al. Evaluation of Vancomycin Use in Late-Onset Neonatal Sepsis Using the Area Under the Concentration-Time Curve to the Minimum Inhibitory Concentration ≥400 Target. Ther Drug Monit. 2015;37(6):756-65.

14. Chen J, Huang X, Yu L, Li J, Yang R, Li L, et al. Vancomycin population pharmacokinetics analysis in Chinese paediatric patients with varying degrees of renal function and ages: development of new practical dosing recommendations. J Antimicrob Chemother. 2023;78(8):2037-51.

15. Downes KJ, Zuppa AF, Sharova A, Neely MN. Optimizing Vancomycin Therapy in Critically Ill Children: A Population Pharmacokinetics Study to Inform Vancomycin Area under the Curve Estimation Using Novel Biomarkers. Pharmaceutics. 2023;15(5).

16. Lv M, Yang P, Zhang S, Wang L, Sun K, Zhao L. Population Pharmacokinetics and Dosage Optimization of Vancomycin in Pediatric Patients with Skin and Soft Tissue Infections, Bone, and Joint Infections. Antimicrob Agents Chemother. 2023;67(1):e0162422.

17. Koedood L, De Haan TR, Hodiamont CJ, Van Haelst IMM, Mathot RAA. Evaluation of a new Dutch guideline for vancomycin dosing and population pharmacokinetics of vancomycin for preterm and term neonates. Pharmaceutisch Weekblad. 2019;154(15):19-23.

18. Sheng XY, Chen CY, Ma LY, Liu YO, Zhou Y, Cui YM. Population pharmacokinetics of vancomycin in Chinese infants Int J Clin Pharmacol Ther. 2017;55(7):558-66.

19. Stockmann C, Olson J, Rashid J, Lubsch L, Young DC, Hersh AL, et al. An Evaluation of Vancomycin Area Under the Curve Estimation Methods for Children Treated for Acute Pulmonary Exacerbations of Cystic Fibrosis Due to Methicillin-Resistant Staphylococcus aureus. J Clin Pharmacol. 2019;59(2):198-205.

20. Egmond PS, Eijkelenburg N, Zwaan C, Mathôt RAA. Evaluation of dosing guidelines and population pharmacokinetics of vancomycin in children with cancer. Pharmaceutisch Weekblad. 2017;152:21-4.

21. Mulla H, Pooboni S. Population pharmacokinetics of vancomycin in patients receiving extracorporeal membrane oxygenation. Br J Clin Pharmacol. 2005;60(3):265-75.

22. Issaranggoon Na Ayuthaya S, Katip W, Oberdorfer P, Lucksiri A. Correlation of the vancomycin 24-h area under the concentration-time curve (AUC(24)) and trough serum concentration in children with severe infection: A clinical pharmacokinetic study. Int J Infect Dis. 2020;92:151-9.

23. Mulubwa M, Griesel HA, Mugabo P, Dippenaar R, van Wyk L. Assessment of Vancomycin Pharmacokinetics and Dose Regimen Optimisation in Preterm Neonates. Drugs R D. 2020;20(2):105-13.

24. Ingrande J, Gutierrez K, Lemmens HJ, Verma A, Nicolau DP, Sutherland CA, Ramamoorthy C. Pharmacokinetics of Cefazolin and Vancomycin in Infants Undergoing Open-Heart Surgery With Cardiopulmonary Bypass. Anesth Analg. 2019;128(5):935-43.

25. Cies JJ, Moore WS, 2nd, Nichols K, Knoderer CA, Carella DM, Chopra A. Population Pharmacokinetics and Pharmacodynamic Target Attainment of Vancomycin in Neonates on Extracorporeal Life Support. Pediatr Crit Care Med. 2017;18(10):977-85.

26. Kato H, Hagihara M, Nishiyama N, Koizumi Y, Mikamo H, Matsuura K, Yamagishi Y. Assessment of optimal initial dosing regimen with vancomycin pharmacokinetics model in very low birth weight neonates. J Infect Chemother. 2017;23(3):154-60.

27. de Hoog M, Schoemaker RC, Mouton JW, van den Anker JN. Vancomycin population pharmacokinetics in neonates. Clinical Pharmacology & Therapeutics. 2000;67(4):360-7.

28. Grimsley C, Thomson AH. Pharmacokinetics and dose requirements of vancomycin in neonates. Arch Dis Child Fetal Neonatal Ed. 1999;81(3):F221-7.

29. Lamarre P, Lebel D, Ducharme MP. A population pharmacokinetic model for vancomycin in pediatric patients and its predictive value in a naive population. Antimicrob Agents Chemother. 2000;44(2):278-82.

30. Wrishko RE, Levine M, Khoo D, Abbott P, Hamilton D. Vancomycin pharmacokinetics and Bayesian estimation in pediatric patients. Ther Drug Monit. 2000;22(5):522-31.

31. Yasuhara M, Iga T, Zenda H, Okumura K, Oguma T, Yano Y, Hori R. Population pharmacokinetics of vancomycin in Japanese pediatric patients. Ther Drug Monit. 1998;20(6):612-8.

32. Burstein AH, Gal P, Forrest A. Evaluation of a sparse sampling strategy for determining vancomycin pharmacokinetics in preterm neonates: application of optimal sampling theory. Ann Pharmacother. 1997;31(9):980-3.

33. Rodvold KA, Gentry CA, Plank GS, Kraus DM, Nickel E, Gross JR. Bayesian forecasting of serum vancomycin concentrations in neonates and infants. Ther Drug Monit. 1995;17(3):239-46.

34. Seay RE, Brundage RC, Jensen PD, Schilling CG, Edgren BE. Population pharmacokinetics of vancomycin in neonates. Clin Pharmacol Ther. 1994;56(2):169-75.

35. Oudin C, Vialet R, Boulamery A, Martin C, Simon N. Vancomycin prescription in neonates and young infants: toward a simplified dosage. Archives of Disease in Childhood - Fetal and Neonatal Edition. 2011;96(5):F365.

36. Zhao W, Lopez E, Biran V, Durrmeyer X, Fakhoury M, Jacqz-Aigrain E. Vancomycin continuous infusion in neonates: dosing optimisation and therapeutic drug monitoring. Arch Dis Child. 2013;98(6):449-53.

37. Anderson BJ, Allegaert K, Van den Anker JN, Cossey V, Holford NH. Vancomycin pharmacokinetics in preterm neonates and the prediction of adult clearance. Br J Clin Pharmacol. 2007;63(1):75-84.

38. Capparelli EV, Lane JR, Romanowski GL, McFeely EJ, Murray W, Sousa P, et al. The influences of renal function and maturation on vancomycin elimination in newborns and infants. J Clin Pharmacol. 2001;41(9):927-34.

39. Chen Y, Wu D, Dong M, Zhu Y, Lu J, Li X, et al. Population pharmacokinetics of vancomycin and AUC-guided dosing in Chinese neonates and young infants. European Journal of Clinical Pharmacology. 2018;74(7):921-30.

40. Colin PJ, Allegaert K, Thomson AH, Touw DJ, Dolton M, de Hoog M, et al. Vancomycin Pharmacokinetics Throughout Life: Results from a Pooled Population Analysis and Evaluation of Current Dosing Recommendations. Clinical Pharmacokinetics. 2019;58(6):767-80.

41. Dao K, Guidi M, André P, Giannoni E, Basterrechea S, Zhao W, et al. Optimisation of vancomycin exposure in neonates based on the best level of evidence. Pharmacol Res. 2020;154:104278.

42. Frymoyer A, Hersh AL, El-Komy MH, Gaskari S, Su F, Drover DR, Van Meurs K. Association between vancomycin trough concentration and area under the concentration-time curve in neonates. Antimicrob Agents Chemother. 2014;58(11):6454-61.

43. Germovsek E, Osborne L, Gunaratnam F, Lounis SA, Busquets FB, Standing JF, Sinha AK. Development and external evaluation of a population pharmacokinetic model for continuous and intermittent administration of vancomycin in neonates and infants using prospectively collected data. Journal of Antimicrobial Chemotherapy. 2019;74(4):1003-11.

44. Jarugula P, Akcan-Arikan A, Munoz-Rivas F, Moffett BS, Ivaturi V, Rios D. Optimizing Vancomycin Dosing and Monitoring in Neonates and Infants Using Population Pharmacokinetic Modeling. Antimicrob Agents Chemother. 2022;66(4):e0189921.

45. Li ZL, Liu YX, Jiao Z, Qiu G, Huang JQ, Xiao YB, et al. Population Pharmacokinetics of Vancomycin in Chinese ICU Neonates: Initial Dosage Recommendations. Front Pharmacol. 2018;9:603.

46. Lo YL, van Hasselt JG, Heng SC, Lim CT, Lee TC, Charles BG. Population pharmacokinetics of vancomycin in premature Malaysian neonates: identification of predictors for dosing determination. Antimicrob Agents Chemother. 2010;54(6):2626-32.

47. Marqués-Miñana MR, Saadeddin A, Peris JE. Population pharmacokinetic analysis of vancomycin in neonates. A new proposal of initial dosage guideline. Br J Clin Pharmacol. 2010;70(5):713-20.

48. Mehrotra N, Tang L, Phelps SJ, Meibohm B. Evaluation of vancomycin dosing regimens in preterm and term neonates using Monte Carlo simulations. Pharmacotherapy. 2012;32(5):408-19.

49. Tseng SH, Lim CP, Chen Q, Tang CC, Kong ST, Ho PC. Evaluating the Relationship between Vancomycin Trough Concentration and 24-Hour Area under the Concentration-Time Curve in Neonates. Antimicrob Agents Chemother. 2018;62(4).

50. Abdel Hadi O, Al Omar S, Nazer LH, Mubarak S, Le J. Vancomycin pharmacokinetics and predicted dosage requirements in pediatric cancer patients. J Oncol Pharm Pract. 2016;22(3):448-53.

51. Alsultan A, Abouelkheir M, Alqahtani S, Aljabri A, Somily AM, Alsubaie S, et al. Optimizing Vancomycin Monitoring in Pediatric Patients. The Pediatric Infectious Disease Journal. 2018;37(9).

52. Avedissian SN, Bradley E, Zhang D, Bradley JS, Nazer LH, Tran TM, et al. Augmented Renal Clearance Using Population-Based Pharmacokinetic Modeling in Critically Ill Pediatric Patients*. Pediatric Critical Care Medicine. 2017;18(9).

53. Chuphan C, Sukarnjanaset W, Puthanakit T, Wattanavijitkul T. Population Pharmacokinetics and Pharmacodynamics of Vancomycin in Pediatric Patients With Various Degrees of Renal Function. J Pediatr Pharmacol Ther. 2022;27(5):419-27.

54. De Cock RF, Allegaert K, Brussee JM, Sherwin CM, Mulla H, de Hoog M, et al. Simultaneous pharmacokinetic modeling of gentamicin, tobramycin and vancomycin clearance from neonates to adults: towards a semi-physiological function for maturation in glomerular filtration. Pharm Res. 2014;31(10):2643-54.

55. Kloprogge F, Hill LF, Booth J, Klein N, Irwin AD, Dixon G, Standing JF. Revising Pediatric Vancomycin Dosing Accounting for Nephrotoxicity in a Pharmacokinetic-Pharmacodynamic Model. Antimicrob Agents Chemother. 2019;63(5).

56. Lanke S, Yu T, Rower JE, Balch AH, Korgenski EK, Sherwin CM. AUC-Guided Vancomycin Dosing in Adolescent Patients With Suspected Sepsis. J Clin Pharmacol. 2017;57(1):77-84.

57. Le J, Bradley JS, Murray W, Romanowski GL, Tran TT, Nguyen N, et al. Improved vancomycin dosing in children using area under the curve exposure. Pediatr Infect Dis J. 2013;32(4):e155-63.

58. Lv CL, Lu JJ, Chen M, Zhang R, Li QC, Chen YY, Liu TT. Vancomycin population pharmacokinetics and dosing recommendations in haematologic malignancy with augmented renal clearance children. J Clin Pharm Ther. 2020;45(6):1278-87.

59. Stockmann C, Sherwin CM, Zobell JT, Lubsch L, Young DC, Olson J, et al. Population pharmacokinetics of intermittent vancomycin in children with cystic fibrosis. Pharmacotherapy. 2013;33(12):1288-96.

60. Zhang T, Cheng H, Pan Z, Mi J, Dong Y, Zhang Y, et al. Desired vancomycin trough concentration to achieve an AUC(0-24) /MIC ≥400 in Chinese children with complicated infectious diseases. Basic Clin Pharmacol Toxicol. 2020;126(1):75-85.

61. Zhao W, Zhang D, Fakhoury M, Fahd M, Duquesne F, Storme T, et al. Population pharmacokinetics and dosing optimization of vancomycin in children with malignant hematological disease. Antimicrob Agents Chemother. 2014;58(6):3191-9.
